# Inhalation of cadmium oxide nanoparticles alters intestinal and pulmonary microbiomes in mice

**DOI:** 10.1007/s00253-026-13970-3

**Published:** 2026-07-28

**Authors:** Chahrazed Mekadim, Daniela Kristeková, Pavel Mikuška, Marcela Buchtová, Jakub Mrázek

**Affiliations:** 1 Laboratory of Anaerobic Microbiology, Institute of Animal Physiology and Genetics, Czech Academy of Sciences, Videnska, 14200 Czech Republic; 2 Laboratory of Molecular Morphogenesis, Institute of Animal Physiology and Genetics, Czech Academy of Sciences, Brno, 60200 Czech Republic; 3 Department of Experimental Biology, Faculty of Science, Masaryk University, Brno, 62500 Czech Republic; 4 Department of Environmental Analytical Chemistry, Institute of Analytical Chemistry, Czech Academy of Sciences, Brno, 60200 Czech Republic

**Keywords:** Gut microbiome, Lung microbiome, Cadmium oxide, Heavy metal, Pollution, Bioaccumulation

## Abstract

**Abstract:**

Exposure to cadmium (Cd), a toxic heavy metal, is a severe threat to organismal health, causing a wide range of pathological alterations in various tissues and organs. Alterations in the composition and function of the gut microbiome have been indicated across numerous animals exposed to Cd. However, the impact of Cd inhalation exposure on the pulmonary microbiome has not been well investigated yet. Therefore, in this study, we investigated the effects of exposure to CdONPs and its clearance on both colonic and pulmonary microbiomes in mice. The diversity of both colonic and pulmonary microbiomes of exposed mice was significantly affected after 9 weeks of CdONPs inhalation. The effects of CdONPs exposure on bacterial composition and function were more pronounced in the colonic microbiome than in the pulmonary microbiome. The clearance was more efficient in the restoration of gut microbiome composition in comparison to the lung microbiome. Moreover, we evaluated a bidirectional interaction between Cd exposure and gut microbiota. *Duncaniella*, *Odoribacter*, and *Pontibacter* were the prominent biomarkers that significantly positively correlated with dysregulated functions in the colonic microbiome of exposed mice. Based on the PICRUSt2 prediction analysis, our results suggested that perturbations in the gut microbiota balance due to Cd exposure were associated with the increase in the proportion level of bacteria with excessive membrane transporters, which may potentially augment the absorption of this metal by intestinal microbiota thereby leading to the accumulation of Cd in intestinal bacteria and the potential alleviation of the Cd toxicity effect. Furthermore, genes related to metal chelators were consistent with the colonic microbiome of exposed mice, suggesting possible promotion of Cd excretion and its eventual fecal elimination. However, these observations derived from 16S rRNA profiling and PICRUSt2 predictions would need to be verified experimentally to establish any functional or mechanistic implications. This could be considered a key factor in determining the intestinal bacterial species able to minimize the toxicity of heavy metals in future therapeutic approaches.

**Key points:**

• *Inhalation of CdO nanoparticles significantly alters both gut and pulmonary microbiome composition and diversity in mice.*

• *Microbiome changes are more pronounced in the gut than in the lungs following inhalation exposure.*

• *Partial recovery of microbiome composition occurs after the clearance period, with greater restoration in the gut than in the lung.*

• *Predicted functional profiles indicate shifts in microbial metabolic potential associated with Cd exposure.*

• *The findings support a potential interaction between inhaled Cd exposure and the gut microbiome, highlighting the relevance of the gut–lung axis.*

**Graphical Abstract:**

**Figure:**
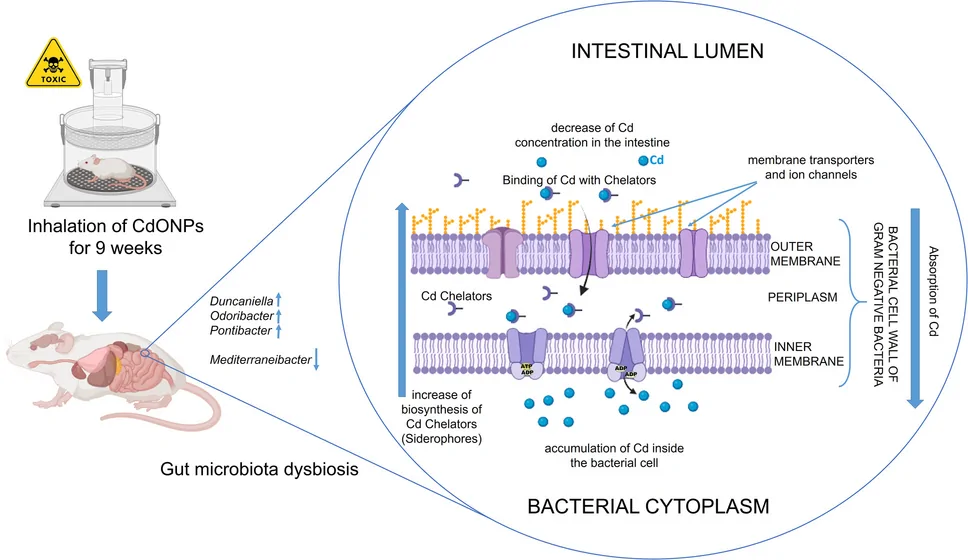

**Supplementary Information:**

The online version contains supplementary material available at 10.1007/s00253-026-13970-3.

## Introduction

Cadmium (Cd) is a toxic heavy metal and one of the most common environmental hazards in agricultural soil, plants, and drinking source water. Cd exposure has become increasingly prevalent through the intake of contaminated air, food, and water or inhalation of tobacco smoke (Rasin et al. 2025). The World Health Organization (WHO) has listed Cd among the ten chemicals of major public health concerns (WHO 2019). Chronic Cd exposure is associated with the risk of various diseases and disorders, including cardiovascular diseases, hepatotoxicity, nephrotoxicity, neurotoxicity, osteoporosis, acute pneumonitis, and lung cancer (WHO 2019; Niture et al. 2021; Bautista et al. 2024; Verzelloni et al. 2024; Rasin et al. 2025; Tang et al. 2025). Cd is accumulated in the human body with a long half-life of 10–35 years (WHO 2019). The accumulated Cd could induce impairments in gut morphology with potentially increased intestinal permeability (He et al. 2020b).

The increased intestinal barrier permeability accompanied by disturbed gut microbiota further exacerbates the toxicity of Cd. The gut microbiota refers to the complex of microorganisms residing in the gastrointestinal tract of host organisms, which plays a key role in the maintenance of host health (Fan and Pedersen 2021). Exposure to Cd was associated with disruptions of the gut microbiota in rodents (Liu et al. 2014, 2020, 2023; Zhang et al. 2015; Zhai et al. 2017a; He et al. 2020a, 2020b; Yang et al. 2021; Li et al. 2022, 2023), frogs (Mu et al. 2018; Ya et al. 2019, 2020; Zheng et al. 2020, 2021), fishes (Chang et al. 2019; Kakade et al. 2020; Xia et al. 2020; Zhang et al. 2020; Yang et al. 2023), shrimps (Duan et al. 2021), mollusks (Jiang et al. 2022; Zhao et al. 2023), insects (Rothman et al. 2019; Šrut et al. 2019; Lee et al. 2020), birds (Wang et al. 2023b; Zhang et al. 2023) and humans (Shao and Zhu 2020; Motta-Romero et al. 2023). Cd exposure induces marked perturbations of gut microbiota characterized by significantly decreased gut bacterial diversity and altered microbial taxa composition. Specifically, gut SCFA-producing bacteria were reduced considerably following the chronic Cd exposure, resulting in decreased SCFAs production in the gut, particularly butyrate (He et al. 2020b; Dai et al. 2022). Previous studies investigating the toxicological effects of Cd on the host’s microbiome showed that gut microbiota disturbance was associated with significant changes in the metabolism of lipids and fatty acids, amino acids, carbohydrates, and bile acids, and perturbation of energy metabolism after exposure to Cd (Li et al. 2019; He et al. 2020b; Chen et al. 2022). The metabolic effects of Cd exposure could lead to life-long metabolic consequences (He et al. 2020a). The gut microbiota alterations caused by Cd toxicity depend on some factors, including exposure time, exposure dose, sex and age (Ba et al. 2017; Nehzomi and Shirani 2025). A bidirectional relationship has been indicated between Cd and the gut microbiota, where Cd can alter the composition and the diversity of the gut microbiota and on the other hand, the gut microbiota regulates the absorption, bioavailability, toxicity or excretion of Cd (Tinkov et al. 2018; Duan et al. 2020; Zhu et al. 2024). Notably, a study revealed that germ-free mice are more susceptible to Cd compared to conventional mice (Breton et al. 2013). Additionally, modulation of gut microbiota via administration of some probiotics with potential protective effects against Cd toxicity may reduce the intestinal absorption of Cd (Zhai et al. 2016; Kumar et al. 2017; Motta-Romero et al. 2023).

Exposure to air pollutants has been linked to an increased risk of lung function impairment. Long-term respiratory exposure to Cd induces lung lesions, lung injury, and immune cell dysregulation in secondary lymphoid organs (Wang et al. 2023a; Prasad et al. 2024). For a long time described as a sterile environment, it is now evident that lungs harbor a complex of microbial communities. In contrast to other microbial populations that reside within other organs, the lung microbiota is characterized by its low biomass, dynamism, and instability. It is becoming increasingly apparent that certain pulmonary disorders can disrupt the host lung microbiota (Natalini et al. 2023). Recent investigations indicated a potential bidirectional gut–lung axis, providing evidence for a crosstalk between lungs and gut microbiome (Özçam and Lynch 2024). However, the effects of Cd inhalation on pulmonary microorganisms have not been well investigated. To date, only one study showed that both microenvironmental factors and pulmonary microbiome were affected under inhalable CdCl_2_ exposure at different doses (Tao et al. 2020), but no studies have examined the influences of CdONPs inhalation on the pulmonary microbiota. Therefore, in this study, we investigate the impacts of CdONPs inhalation on the gut and lung microbiota in order to identify their biomarkers and functional signatures related to alterations in intestinal and pulmonary microbiomes’ diversity, composition, and functionality induced by exposure to inhalable CdO nanoparticles.

## Materials and methods

### Animals and their exposure to CdONPs

Adult female mice (ICR strain), 6–8 weeks old (average weight of approximately 24 g) at the beginning of the inhalation experiment, were obtained from the Animal Facility of Masaryk University (Brno, Czech Republic). Animals were allowed to acclimate to laboratory conditions for 1 week before the start of inhalation experiments, and they had a commercial diet and water available ad libitum. Initially, animals originated from different breeding cages. Prior to the experiment, they were mixed together during the acclimatization period and then randomly assigned to experimental cages/groups. This approach was intentionally used to minimize potential cage and breeding-origin effects on microbiome composition, ensuring that any baseline microbiome differences were distributed across groups rather than confounded with treatment. We used only female mice in this study (details described in Dumková et al. 2025). Details of all mice are listed in Table S1.

**Fig. 1:**
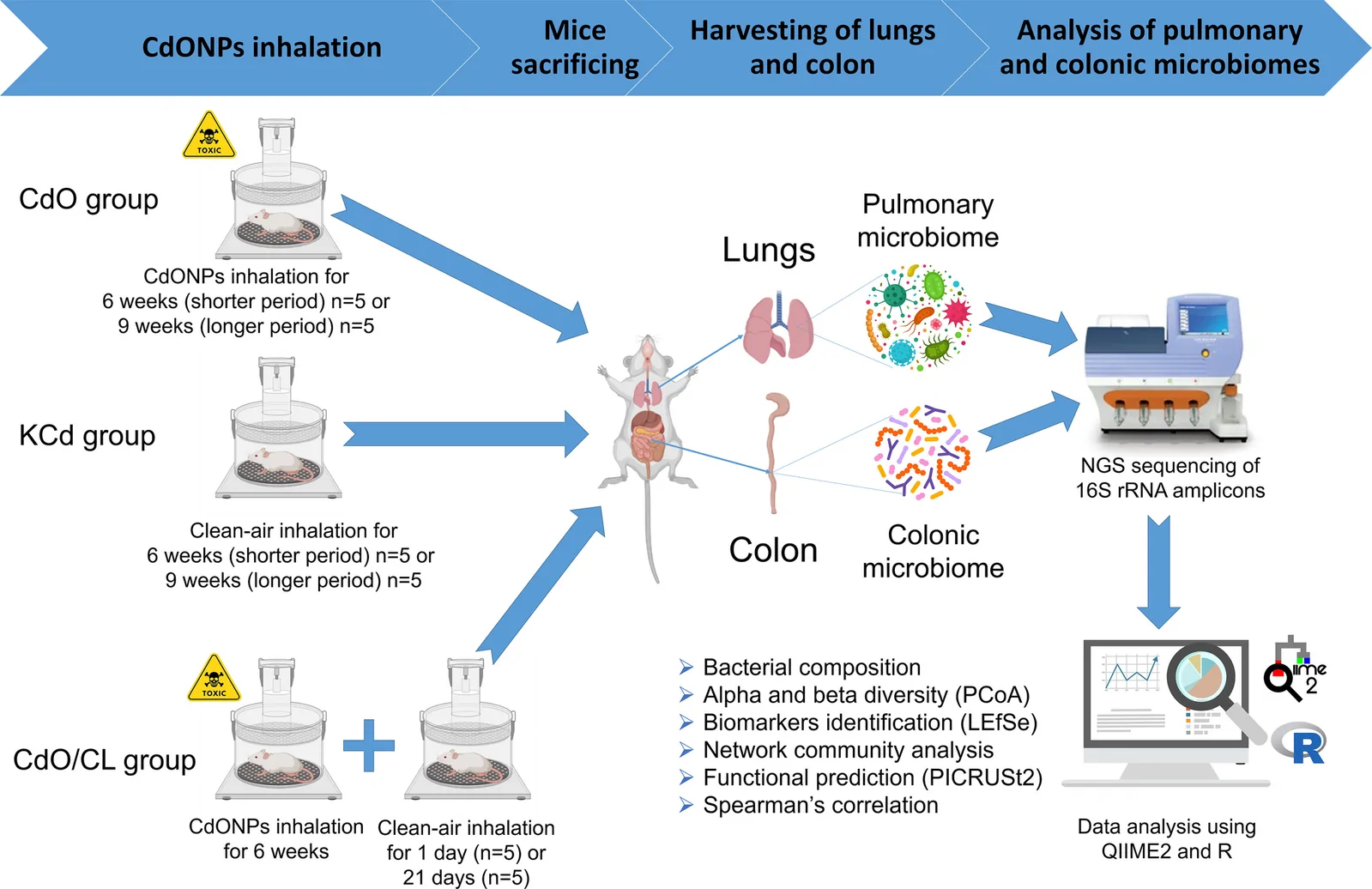
The experimental schema outlines the sequential steps undertaken in this study to assess the impact of exposure to CdONPs on pulmonary and colonic microbiomes. There are three groups of mice in this experiment: (1) CdO group: Mice were continuously exposed to CdONPs for 6 or 9 weeks (CdO/6w+1d (*n* = 5) or CdO/9w (*n* = 5)) in a whole-body inhalation chamber, (2) KCd group: Control animals were exposed to the clean air 6 or 9 weeks (KCdO/6w+1d (*n* = 5) or KCd/9w (*n* = 5)), and (3) CdO/CL group: Mice were exposed to CdONPs for 6 weeks and then transferred to clean air for post-exposure periods for 1 or 21 days (CdO/CL/6w+1d (*n* = 5) or CdO/CL/6w+21d (*n* = 5)). At the end of the exposure period, the mice were sacrificed by cervical dislocation. Samples of lungs and colon were collected and used for analysis of the changes in the pulmonary and colonic microbiomes

Mice were continuously exposed to CdONPs for up to 9 weeks (24 h/day, 7 days/week) in a whole-body inhalation chamber. Generation of nanoparticles and their characterization were described previously (Dumková et al. 2025). Control animals were exposed to the same air as the experimental group, but without NPs (KCd group). In addition to the CdO-exposed group and the clean air control group, a third group was used, which was referred to as the clearance group (CdO/CL). Mice of this group were exposed to CdONPs for 6 weeks and then transferred to clean air for post-exposure periods for 1 or 21 days (i.e., CdO/CL/6w+1d or CdO/CL/6w+21d, labeled as clearance group). All these animals were used to evaluate the microbiome in exposed animals as well as during the clearance period. At the end of the exposure period, the mice were sacrificed by cervical dislocation, samples of lungs and colon were collected and frozen before further analyses. The study workflow is elucidated in the experimental scheme (Fig. 1).

### DNA isolation and sequencing of 16S rRNA amplificons

DNA was extracted from colon and lung samples of control and exposed mice using the QIAamp PowerFecal Pro DNA Kit (QIAGEN, Germany) following the manufacturer’s instructions. The extracted DNA served as a template for amplifying the 16S rRNA gene. The purified amplicons of the V4 region of the 16S rRNA gene (300 bp) were used to construct sequencing libraries with the NEBNext®Fast DNA Library Prep Kit (New England Biolabs, USA) as described by Milani et al. (2013) (Milani et al. 2013). The barcode-ligated purified libraries were subsequently sequenced on the Ion Torrent platform (Thermo Fisher Scientific, USA) (Mekadim et al. 2022).

### Microbiome analysis and statistical tests

Bacterial 16S rRNA gene sequences were acquired in FASTQ format and processed using QIIME 2 (version 2024.2; Bolyen et al. 2019). Sequence quality control, including filtering, chimera removal, and length trimming, was conducted via the DADA2 pipeline (Callahan et al. 2016), resulting in high-resolution amplicon sequence variants (ASVs). Taxonomic classification of ASVs was performed using the rEGEN-B database (rrn operons Extracted from GENomes of Bacteria; Dubois et al. 2022), a newly curated reference resource.

Rarefaction was applied to normalize sequencing depth across samples. Alpha-diversity metrics, including Pielou’s evenness, Shannon, Chao1, and Simpson indices, were calculated and statistically evaluated using the Kruskal–Wallis test. Beta-diversity was assessed through Principal Coordinate Analysis (PCoA) based on Bray–Curtis dissimilarity. Visualization of alpha-diversity boxplots and 2D PCoA plots was performed in R using the ggplot2 package, with 95% confidence ellipses delineating group distributions. Group-level dissimilarities were statistically tested using permutational multivariate analysis of variance (PERMANOVA) with pairwise Adonis comparisons, employing a Bray–Curtis distance matrix and 999 permutations. A *P*-value threshold of < 0.05 was considered significant.

To identify genera with significantly different relative abundances between groups, the LEfSe (Linear Discriminant Analysis Effect Size) algorithm was applied using the Kruskal–Wallis and pairwise Wilcoxon’s tests, with an alpha cutoff of 0.05 and an LDA score threshold of 2.0, implemented via the lefser package in R (Khleborodova et al. 2024).

Functional pathway prediction was carried out using PICRUSt2 (Douglas et al. 2020), referencing KEGG level 3 annotations. Differentially abundant pathways and bacterial genera were identified using the STAMP software (Parks et al. 2014), applying a two-sided Welch’s *t*-test without correction, with a 95% confidence interval and significance set at *P* < 0.05.

The correlation analysis between identified biomarkers and predicted functional pathways was carried out using the Spearman’s rank correlation test (Spearman’s rank correlation test, *P* < 0.05). Multiple‑testing correction was performed using the Benjamini–Hochberg method. Raw *P-*values, Spearman’s correlation coefficient, and BH-adjusted values are reported in Supplementary Table S8 for completeness. Co-occurrence bacterial network analysis of both pulmonary and intestinal microbiomes was performed using the NetCoMi (Peschel et al. 2021) package from ASVs data at the genus level based on SPRING association via the “netConstruct” function. Differential network association analysis was conducted using the “diffnet” function from NetCoMi with permutation testing and adjusted with “lfdr”.

## Results

Bacterial community composition and diversity were analyzed using 16S rRNA gene sequencing in a total of 60 samples, comprising 30 colon and 30 lung samples collected from control, CdONPs-exposed, and clearance groups (Fig. 1; Table S1). Samples were obtained at defined time points representing exposure (9 weeks) and post-exposure recovery periods (1 day and 21 days after cessation of exposure). Across all samples, a total of 4,121,107 sequence reads were obtained, with a mean read length of 291 bp. These data were used to assess taxonomic composition, microbial diversity, and predicted functional profiles associated with CdONPs exposure and subsequent clearance in both intestinal and pulmonary microbiomes.

### Effects of CdONPs exposure on the lung and gut bacterial diversity of mice

**Fig. 2:**
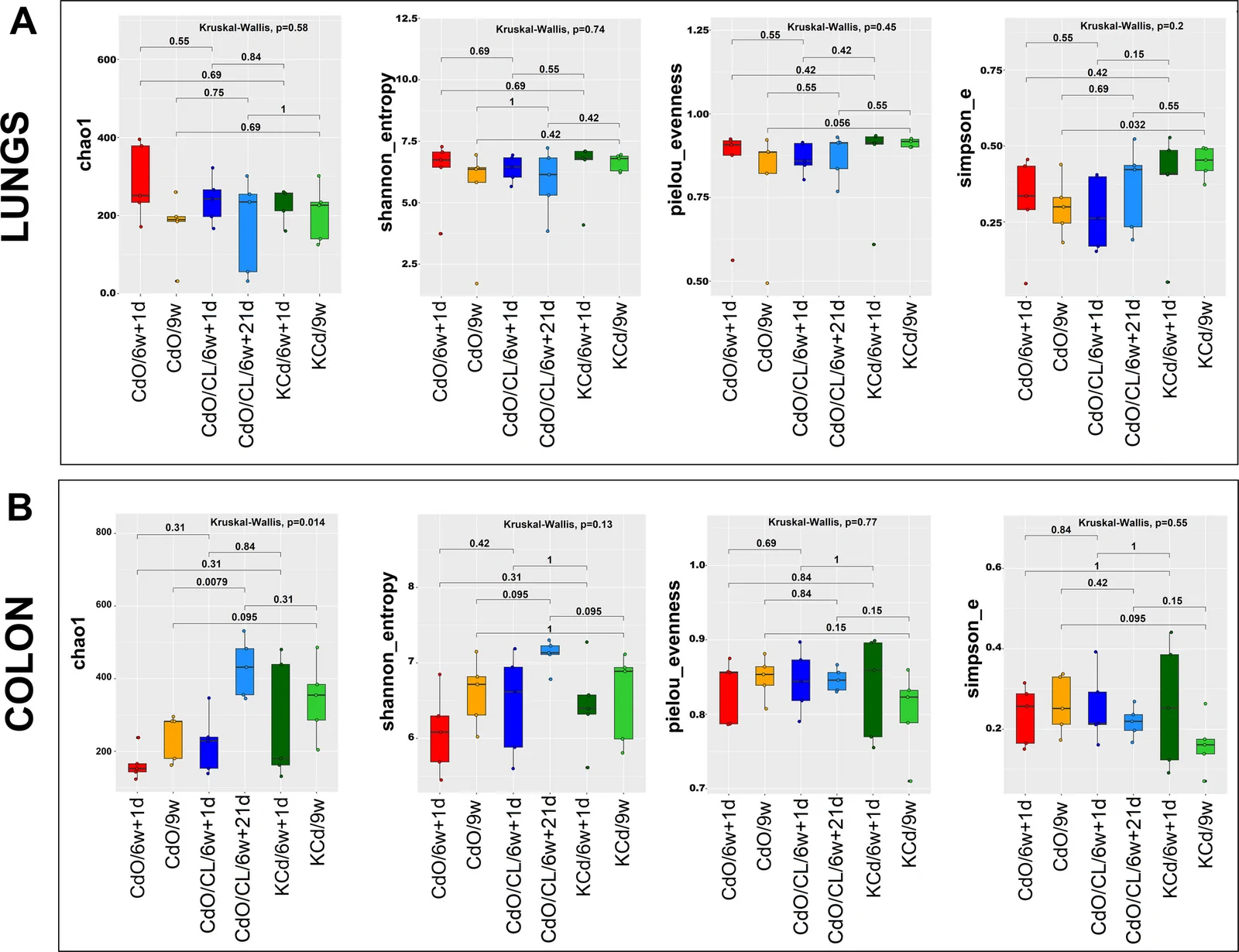
Effect of CdONPs exposure on the pulmonary and gut bacterial diversity. Boxplots illustrating Chao1, Shannon, Simpson, and Pielou_evenness diversity indices in the bacterial community of lungs (**A**) and colon (**B**) of different mice groups after 1 day and 21 days of clearance following 6 weeks of inhalation with CdONPs or just exposure to nanoparticles for the whole period. *P* < 0.05 was considered statistically significant based on the Kruskal–Wallis test (size sample for each group: *n* = 5)

Alpha-diversity analysis using Pielou’s evenness, Chao1, Shannon, and Simpson indices showed no significant difference in the richness and the evenness of gut microbiome diversity between different treatments (KCd/6w+1d, CdO/6w+1d, CdO/CL/6w+1d) in both colon microbiome and lung microbiome after 1 day following 6 weeks of exposure to CdONPs (Fig. 2). After 21 days following 6 weeks of inhalation with CdONPs, significant differences were observed in the lung microbiome between KCd/9w and CdO/9w mice groups using Simpson’s index (*P* = 0.032) (Fig. 2A) and in the colon microbiome between CdO/9w and CdO/CL/6w+21d exposure using Chao1 index with *P* = 0.0079 (Fig. 2B).

**Fig. 3:**
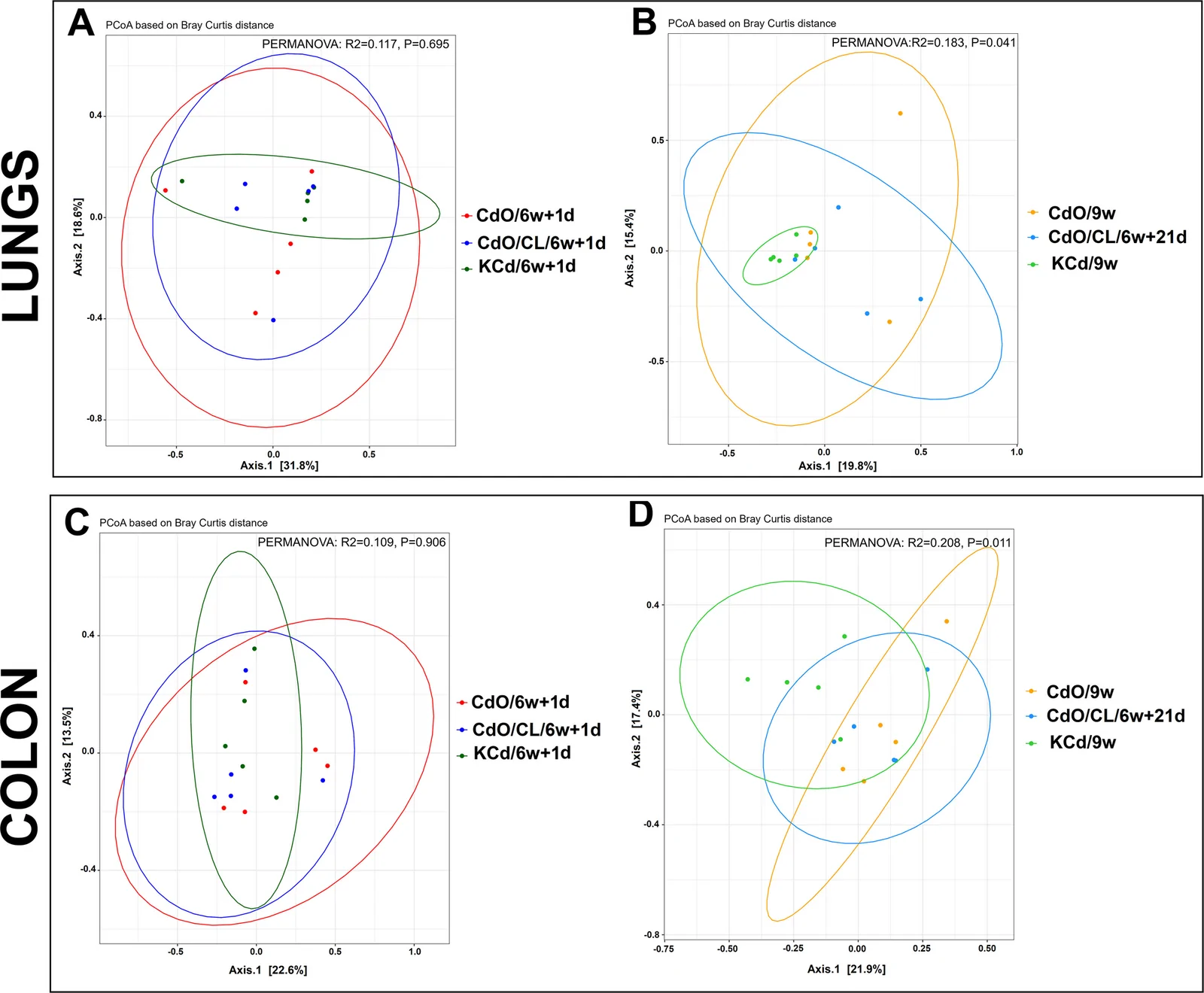
Effect of CdONPs exposure on the beta diversity of the pulmonary and gut bacterial diversity in mice. Principal Coordinate Analysis (PCoA) plots based on the Bray–Curtis distance analyses revealed distinct clusters in the microbiome of lungs (**A** and **B**) and colon (**C** and **D**) after 6w+1d (**A** and **C**) and 6w+21d (**B** and **D**) CdONPs exposition. *P* < 0.05 was considered statistically significant based on pairwise PERMANOVA with 999 permutations, significant differences were observed in lung microbiome (KCd/9w vs CdO/9w, *p = 0.043; KCd/9w vs CdO/CL/6w+21d, *p = 0.013) and in colonic microbiome (KCd/9w vs CdO/9w, *p = 0.016; KCd/9w vs CdO/CL/6w+21d, *p = 0.015) (size sample for each group, *n* = 5)

Beta-diversity analysis using PCoA based on Bray–Curtis distance showed no significant difference between groups of samples in both organs after 1 day followed by 6 weeks of inhalation with CdONPs (Fig. 3A and C; Table S2A and C). However, significant differences were revealed between the control group (KCd/9w) and both exposed groups (CdO/9w, CdO/CL/6w+21d) in both organs after 21 days following 6 weeks of inhalation with Cd (Fig. 3B and D; Table S2B and D).

### Effects of CdONPs exposure on the lungs and gut bacterial composition

**Fig. 4:**
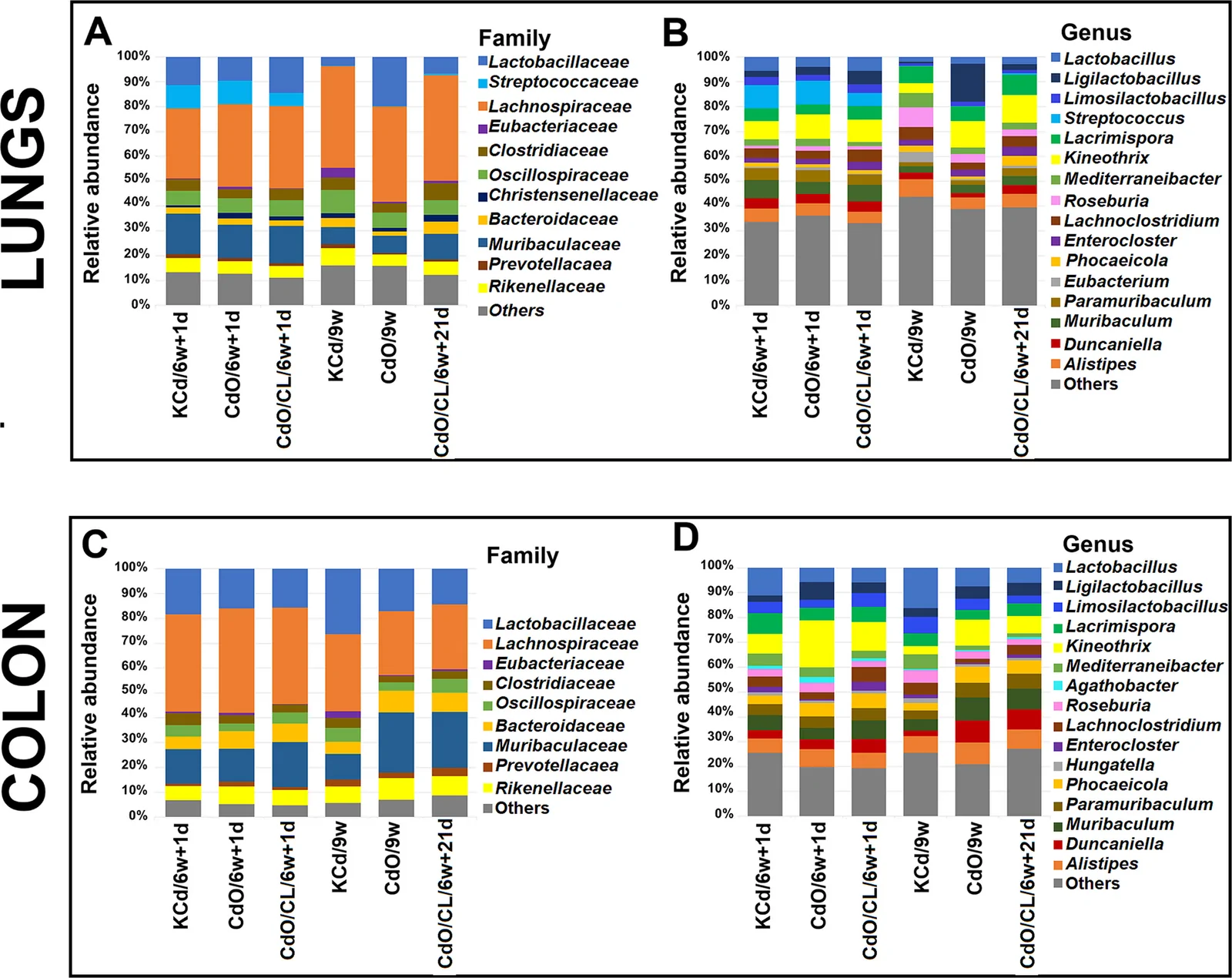
Effect of CdONPs exposure on the pulmonary and gut microbiota composition. Relative abundance of bacterial communities at family (**A** and **C**) and genus (**B** and **D**) levels in microbiome of lungs (**A** and **B**) and colon (**C** and **D**) of mice exposed to CdOPNs for 6w+1d or 6w+21d period (size sample for each group, *n* = 5)

The relative abundance of bacterial composition was assessed at phylum, family, and genus levels to investigate the effects of Cd inhalation and clearance on the lungs and gut microbiota of exposed mice compared to control (Fig. 4; Fig. S1 and Tables S3, S4, S5).

At the phylum level (Fig. S1), three bacterial phyla were identified in the lung microbiota (Fig. S1A): *Bacillota* (formerly named *Firmicutes*), *Bacteroidota* (formerly named *Bacteroidetes*), *Pseudomonadota* (formerly named *Proteobacteria*); however, only *Bacillota* and *Bacteroidota* were the most abundant phyla in the colon microbiota of all mice (Fig. S1B). The relative abundance analyses of bacteria, at family (Fig. 4A and C) and genus (Fig. 4B and D) levels, uncovered the bacterial composition in the microbiome of control (KCd) and both exposed groups (CdO and CdO/CL) in each organ: lungs (Fig. 4A and B) and colon (Fig. 4C and D). *Bacillota* was found to be the dominant phylum in all samples. The main families of *Bacillota* were *Lachnospiraceae*, *Oscillospiraceae*, and *Lactobacillaceae.* The phylum *Bacteroidota* was mainly represented by the families of *Muribaculaceae* and *Bacteroidaceae* (Fig. 4A and C).

At the genus level (Fig. 4B and D), *Lactobacillus*, *Ligilactobacillus*, *Limosilactobacillus*, *Lacrimispra*, *Kineothrix*, *Roseburia*, *Alistipes*, *Paramuribaculum*, and *Muribaculum* were the most abundant bacterial genera in the lung and colon microbiota of all studied mice. Particularly, *Streptococcus* was significantly higher only in the lung microbiota of mice after a shorter time period of inhalation with Cd. Markedly, the abundance of Lactobacillaceae increased in the pulmonary microbiome (19,80%) and decreased in the colonic microbiome (17,06%) of mice exposed to CdONPs for a longer period (CdO/9w) than in their control counterpart (KCd/9w) (3,61% and 26,38% respectively) (Fig. 4A and C; Tables S3 and S4).

**Fig. 5:**
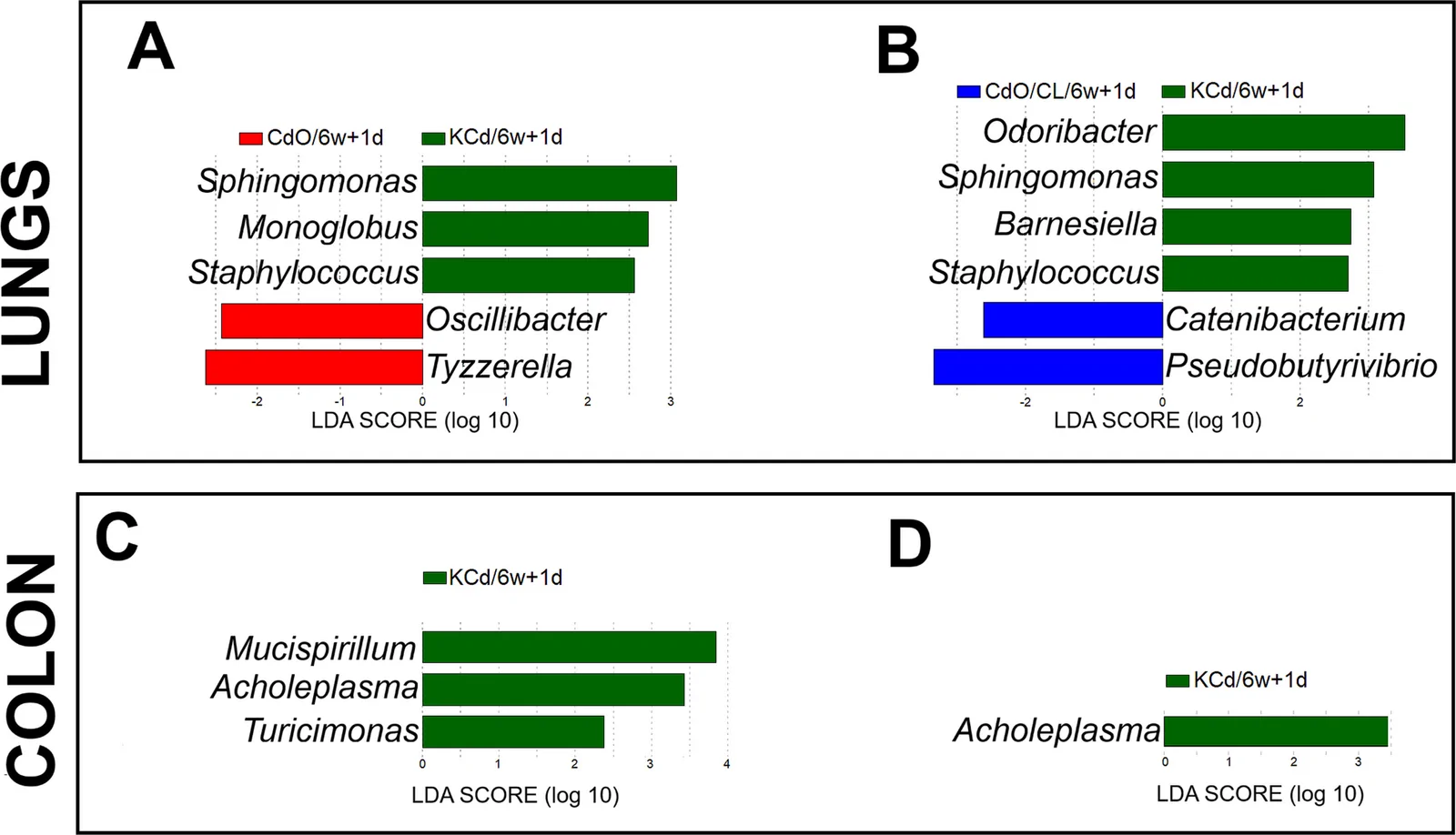
Bacterial markers related to CdONPs exposure in the pulmonary and gut microbiome for a shorter time period. Linear discriminant analysis effect size (LEfSe) of taxa at genus level in the microbiome of lungs (A, B) and colon (C, D) of mice exposed to CdONPs for a shorter time (6w+1d time period) with alpha values of 0.05 and a threshold value of 2.0 (size sample for each group, *n* = 5)

**Fig. 6:**
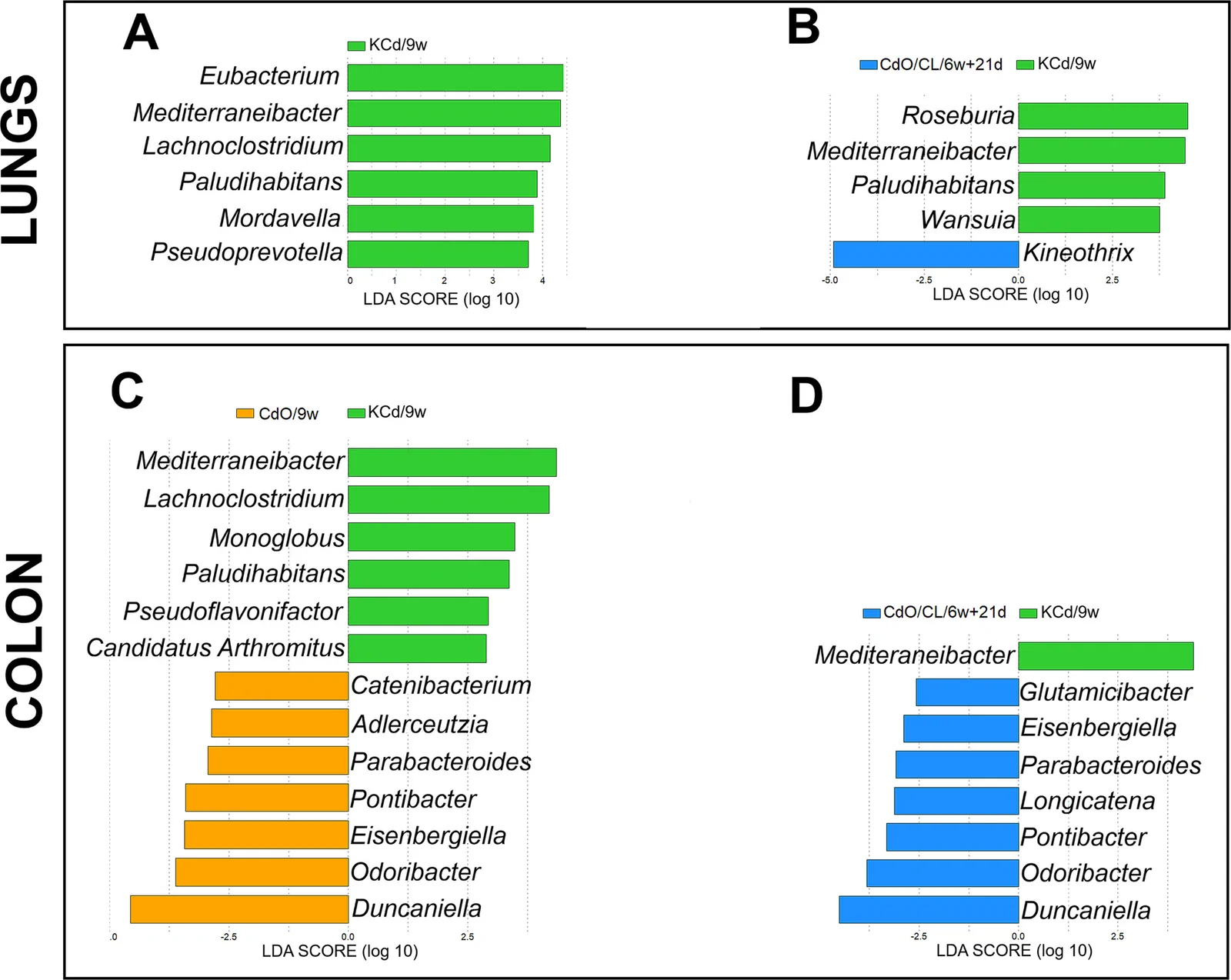
Bacterial markers related to CdONPs exposure in the pulmonary and gut microbiome for a longer time period. Linear discriminant analysis effect size (LEfSe) of taxa at genus level in the microbiome of lungs (**A** and **B**) and colon (**C** and **D**) of mice exposed to CdONPs for a longer time (6w+21d time period) with alpha values of 0.05 and a threshold value of 2.0 (size sample for each group, *n* = 5)

LEfSe was used to identify the features at the genus level that were differentially abundant between the control mice and exposed mice to assess the gut bacterial marker specific to each treatment (Figs. 5 and 6). After 6w+1d inhalation of CdONPs, *Tyzzerella* and *Oscillibacter* were bacterial markers in the lung microbiota of mice exposed to CdO (Fig. 5A) and *Catenibacterium* and *Pseudobutyrivibrio* were bacterial markers in the lung microbiota of mice exposed to nanoparticles and followed their clearance (Fig. 5B) compared to the lung microbiota in control mice. No specific bacterial markers were determined in the gut microbiota of both exposed groups (CdO/6w+1d and CdO/CL/6w+1d) (Fig. 5C and D). *Sphingomonas* and *Staphylococcus* were the common bacterial markers in the lung microbiome of the control group (KCd/6w+1d) (Fig. 5A and B), while *Acholeplasma* was the only common bacterial marker in the colonic microbiome of the control group (KCd/6w+1d) (Fig. 5C and D).

No significant biomarker was identified in the lung microbiota of mice exposed to CdONPs for 9 weeks (Fig. 6A), and *Kineothrix* was the only bacterial marker uncovered in the lung microbiota of the clearance group (CdO/CL/6w+21d) (Fig. 6B) compared to the lung microbiota of control mice. Seven bacterial markers were identified in the colonic microbiome of CdONPs-exposed mice for 9 weeks (Fig. 6C and D). *Duncaniella*, *Odoribacter*, *Eisenbergiella*, *Pontibacter*, and *Parabacteroides* were the common bacterial markers in the colonic microbiome of both CdO-exposed groups (CdO/9w, CdO/CL/6w+21d) (Fig. 6C and D). *Mediterraneibacter* was the common bacterial marker in the pulmonary and colonic microbiomes of the control group (KCd/9w) (Fig. 6). Likewise, the colonic microbiome exhibited a higher number of significantly altered genera compared with the lung microbiome in mice exposed to CdONPs for 9 weeks (CdO/9w) (Table S5), consistent with the LEfSe‑based pattern shown in Fig. 6.

In summary, the alteration in bacterial composition was more prominent in the colonic microbiome of mice exposed to CdONPs for 9 weeks.

### Effects of CdONPs exposure on the co-occurrence bacterial network in lungs and gut

Differential networks were inferred to reveal taxa that were involved either in co-abundance (positive correlation) or in co-exclusion (negative correlation) in exposed mice (CdO and CdO/CL) versus control mice in both organs (lung and colon) after 1 day or 21 days followed by 6 weeks of CdONPs inhalation. Bacterial correlation network analysis exhibited clearly distinct differences in bacterial taxa co-occurrence in exposed mice versus control mice in both organs (lung and colon). *Alistipes*, *Blautia*, *Dorea*, *Duncaniella*, *Kineothrix*, *Lactobacillus*, *Prevotella*, *Qiania*, *Roseburia*, and *Wansuia* are among the main central genera that cross-correlate with several bacterial genera (Figs. S2 andS3).

*Lactobacillus* showed positive cross-correlation with *Muribaculum* in the pulmonary microbiome of the control (KCd/6w+1d) and clearance group (CdO/CL/6w+1d), while negative cross-correlations were uncovered in the group exposed to CdONPs for the whole period (CdO/6w+1d). *Odoribacter* and *Sphingomonas* displayed positive cross-correlations with *Duncaniella* and *Candidatus Phytoplasma*, respectively, in the pulmonary microbiome of the control mice (KCd/6w+1d) and negative cross-correlations in CdONP mice (CdO/6w+1d) (Fig. S2A and B). *Acholeplasma* showed positive cross-correlation with *Ligilactobacillus* in the colonic microbiome of both exposed groups (CdO/6w+1d and CdO/CL/6w+1d) and negative cross-correlation in the control mice (KCd/6w+1d). *Mucispirillum* positively cross-correlated with *Muribaculum* and *Duncaniella* in the colonic microbiome of exposed mice (CdO/6w+1d) and negatively cross-correlated with the control mice (KCd/6w+1d) (Fig. S2C and D).

*Roseburia* displayed positive cross-correlation with *Clostridium* in the pulmonary microbiome of the clearance group (CdO/CL/6w+21d). *Wansuia* showed positive cross-correlation with *Limosilactobacillus* in the pulmonary microbiome of the control group (KCd/9w) and negative cross-correlations with both CdONPs-exposed mice (CdO/9w and CdO/CL/6w+21d). Negative cross-correlation was determined for *Mediterraneibacter* with *Anaerotruncus* and *Limnospira* in the pulmonary microbiome of both exposed groups (CdO/9w and CdO/CL/6w+21d), respectively, and positive cross-correlation with the control mice (KCd/9w) (Fig. S3A and B). *Duncaniella* exhibited positive cross-correlation with *Ligilactobacillus* and *Amidibacterium* in the colonic microbiome of both exposed groups (CdO/9w and CdO/CL/6w+21d), respectively. *Odoribacter* showed negative cross-correlation with *Dehalobacterium* in the colonic microbiome of exposed mice (CdO/9w) and positive cross-correlation with *Alistipes* in the colonic microbiome of the clearance group (CdO/CL/6w+21d). Negative cross-correlation was found between *Eisenbergiella* and *Adlercreutzia* in the colonic microbiome of exposed mice (CdO/9w). *Parabacteroides* and *Pontibacter* showed negative cross-correlation with *Lachnoclostidium* and *Hominenteromicrobium*, respectively, in the colonic microbiome of both exposed groups (CdO/9w and CdO/CL/6w+21d). Negative cross-correlation was determined between *Mediterraneibacter* and *Wansuia* in the colonic microbiome of both exposed groups (CdO/9w and CdO/CL/6w+21d) and positive cross-correlation in the control mice (KCd/9w) (Fig. S3C and D).

In addition to its effects on bacterial composition and diversity, exposure to CdONPs significantly reshapes the structure of the bacterial co-occurrence network, indicating broader disruptions in microbial community dynamics.

### Effects of CdONPs exposure on the functional profile of lung and gut microbiota

PICRUSt2 was used to determine the effect of CdONPs exposure on functional pathways in the lung and intestinal microbiota of exposed mice. Significant differences were determined in functional profiles of microbiomes of both analyzed organs in exposed mice compared to control mice (Figs. S4–S7; Tables S6, S7). KEGG functional pathway analysis uncovered many microbial functional genes related to “metabolism,” “environment information processing,” “genetic information processing,” “cellular processes,” “organismal systems,” and “human diseases” in lung and gut microbiota of mice. The influence of Cd exposure was more prominent in the colonic microbiome of exposed mice (CdO/9w), consistent with multiple predicted functional pathways (Fig. S6B).

### Correlations between differentially abundant genera and altered functional pathways induced by CdONPs inhalation in lung and gut microbiota

**Fig. 7:**
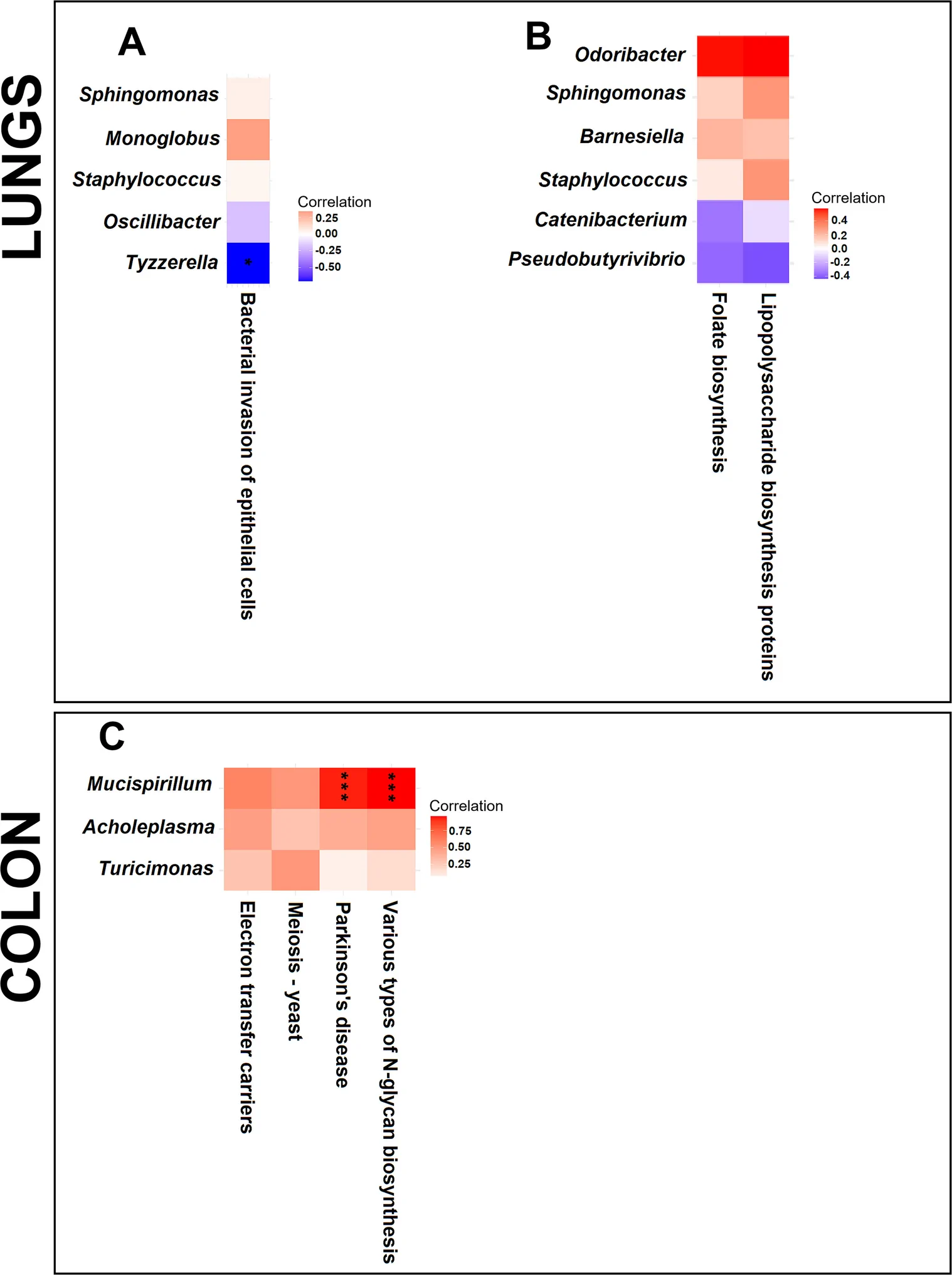
Correlations between identified biomarkers and perturbed functions in the pulmonary and gut microbiome in mice exposed to CdONPs for a shorter time period. The correlation plot summarizes associations between the bacterial genera markers and altered functions in the microbiome of lungs (**A** and **B**) and colon (**C**) of mice exposed to CdONPs for a shorter time (6w+1d period). Red indicates positive correlations; blue indicates negative correlations; color intensity reflects correlation strength; *P < 0.05; **P < 0.01; ***P < 0.001 (size sample for each group, *n* = 5)

**Fig. 8:**
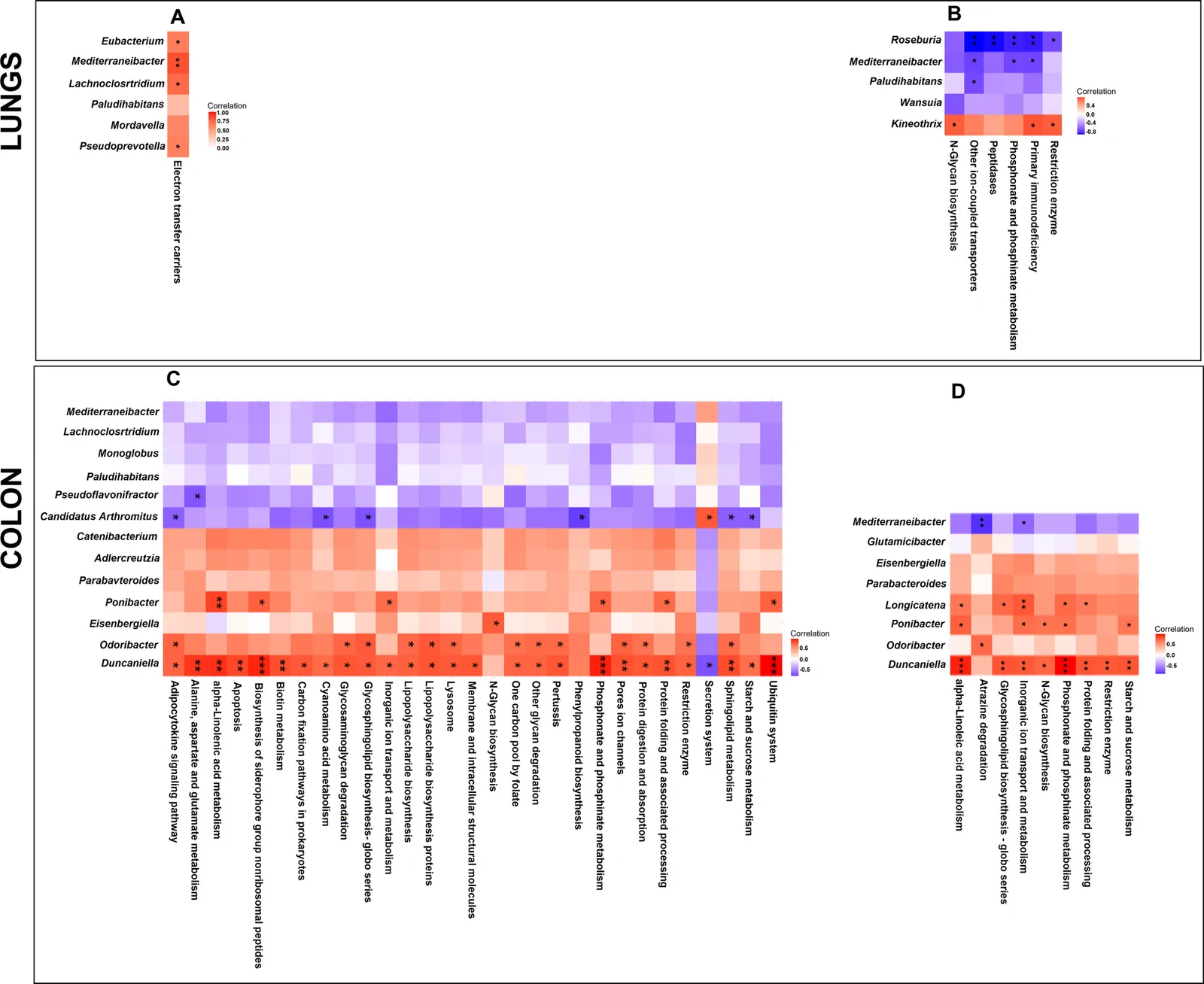
Correlations between identified biomarkers and perturbed functions in the pulmonary and gut microbiome in mice exposed to CdONPs for a longer time period. The correlation plot summarizes associations between the bacterial genera markers and altered functions in the microbiome of lungs (**A** and **B**) and colon (**C** and **D**) of mice exposed to CdONPs for a longer time (6w+21d period). Red indicates positive correlations; blue indicates negative correlations; color intensity reflects correlation strength, *P < 0.05; **P < 0.01; ***P < 0.001 (size sample for each group: *n* = 5)

Spearman’s correlation analysis for significantly different functions and microbes was performed to obtain the relationships between altered functional pathways and identified bacterial markers specific for each group in pulmonary and colonic microbiomes of mice (Figs. 7 and 8). Because this analysis involved a small, pre‑filtered set of taxa, multiple‑testing corrections can become overly conservative. Adjusted *p*‑values (Benjamini–Hochberg) are provided in Supplementary Table S8 for transparency, but the main interpretation focuses on raw *p*‑values and Spearman’s correlation coefficient because most previously significant associations were substantially reduced or became non‑significant after correction.

Gene encoding “bacterial invasion of epithelial cells” was significantly negatively correlated with *Tyzzerella* in the pulmonary microbiome of control mice (KCd/6w+1d) (Fig. 7A). *Mucispirillum* was significantly positively correlated with “various types of *N*-glycan biosynthesis” and “Parkinson’s disease” pathways in the colonic microbiome of control mice (KCd/6w+1d) (Fig. 7C).

“Electron transfer carriers” functional pathway was significantly positively correlated with *Mediterraneibacter*, *Eubacterium*, *Lachnoclostridium* and *Pseudoprevotella* in the pulmonary microbiome of the control mice (KCd/9w) (Fig. 8A). *Roseburia* bacterial marker in the lung microbiome of control mice (KCd/9w) was significantly negatively correlated with “other ion-coupled transporters,” “peptidases,” “phosphonate and phosphinate metabolism,” “primary immunodeficiency,” and “restriction enzyme,” which were relevant to the pulmonary microbiome of the clearance group (CdO/CL/6w+21d). “Other ion-coupled transporters” pathway was also significantly negatively correlated with *Mediterraneibacter* and *Paludihabitans* bacterial markers in the lung microbiome of control mice (KCd/9w)*. Mediterraneibacter* was also significantly negatively correlated with “phosphonate and phosphinate metabolism” and “primary immunodeficiency” pathways. *Kineothrix* bacterial marker in the pulmonary microbiome of the clearance group (CdO/CL/6w+21d) was significantly positively correlated with “*N*-glycan biosynthesis,” “primary immunodeficiency,” and “restriction enzyme” pathways in the pulmonary microbiome of the clearance group (CdO/CL/6w+21d) (Fig. 8B).

*Candidatus Athromitus*, bacterial marker in the colonic microbiome of control mice (KCd/9w) was significantly positively correlated with the “secretion system” pathway predicted in the colonic microbiome of control mice (KCd/9w) and significantly negatively correlated with pathways predicted in the colonic microbiome of exposed mice (CdO/9w): “adipocytokine signaling pathway,” “cyanoamino acid metabolism,” “glycosphingolipid biosynthesis,” “phenylpropanoid biosynthesis,” “sphingolipid metabolism,” and “starch and sucrose metabolism” (Fig. 8C). *Duncaniella* bacterial marker in the colonic microbiome of exposed mice (CdO/9w and CdO/CL/6w+21d) was significantly positively correlated with almost all pathways predicted in the colonic microbiome of both exposed groups (CdO/9w and CdO/CL/6w+21d) (Fig. 8C and D). *Eisenbergiella* bacterial marker in the colonic microbiome of exposed mice (CdO/9w) was significantly positively correlated with only one pathway “*N*-glycan biosynthesis,” which was predicted in the colonic microbiome of exposed mice (CdO/9w) (Fig. 8C). *Odoribacter* bacterial marker in the colonic microbiome of exposed mice (CdO/CL/6w+21d) was significantly positively correlated with only one pathway “atrazine degradation,” which was predicted in the colonic microbiome in the clearance group (CdO/CL/6w+21d) (Fig. 8D). *Mediterraneibacter* bacterial marker in the lung microbiome of control mice (KCd/9w) was significantly negatively correlated with the “atrazine degradation” and “inorganic ion transport and metabolism” pathways, which were predicted in the colonic microbiome of the clearance group (CdO/CL/6w+21d) (Fig. 8D).

In summary, the colonic microbiome of mice exposed to CdONPs for a longer period exhibited more predicted functions, characterized by different microbial metabolic pathways.

## Discussion

The crucial role of the gut and lung microbiota in human health has been well established (Fan and Pedersen 2021; Natalini et al. 2023; Özçam and Lynch 2024), and exposure to heavy metals such as cadmium (Cd) can cause significant damage to various tissues and organs, including the lungs and intestines (Wang et al. 2023a; Rasin et al. 2025). Several studies have revealed that environmental exposure to Cd could generate adverse impacts on intestinal physiology and alter gut microbiota homeostasis (Liu et al. 2014; Tinkov et al. 2018; Yang et al. 2021; Li et al. 2022; Tao et al. 2024). Despite extensive investigations of Cd toxicity on the gut microbiota of various model animals, there remains a lack of comprehensive research on the impact of this heavy metal on the pulmonary microbiome. Our study, therefore, explored the influence of CdONPs exposure on the bacterial diversity, composition and functional pathways of both gut and lung microbiomes of mice.

The effects of CdONPs exposure were more pronounced in the colonic microbiome than in the pulmonary microbiome, despite inhalation being the primary route of exposure. Although most studies on Cd-microbiome interactions have focused on oral exposure (Natalini et al. 2023; Özçam and Lynch 2024), several physiological mechanisms may explain how inhaled CdONPs influence the gut microbiota. First, particles deposited in the respiratory tract can be transported via the mucociliary escalator to the pharynx and subsequently swallowed, providing a direct route for Cd to enter the gastrointestinal tract and interact with the intestinal microbiome (Enaud et al 2020). Second, pulmonary exposure may induce systemic effects through the gut–lung axis, a bidirectional communication network linking respiratory and intestinal systems (Budden et al. 2016; Dumas et al. 2018; He et al. 2017). Cd-induced inflammation, oxidative stress, and immune modulation in the lungs may alter intestinal barrier integrity and immune homeostasis, thereby contributing to shifts in microbial composition (Oberdörster et al. 2005). These mechanisms are not mutually exclusive and may act in parallel. Although our study does not directly assess these pathways, their consideration provides a biologically plausible framework for interpreting the observed gut microbiome alterations following inhalation exposure.

Alpha and beta diversity analyses uncovered that CdONPs exposure and clearance did not affect the diversity of both colonic and pulmonary microbiomes of exposed mice (CdO/6w+1d and CdO/CL/6w+1d) in contrast to their control counterpart KCd/6w+1d. However, CdONPs exposure and clearance significantly affected the diversity of both colonic and pulmonary microbiomes of exposed mice (CdO/9w and CdO/CL/6w+21d) for a longer period in contrast to their control counterpart KCd/9w. This could be due to the fact that Cd toxicity depends on factors such as exposure time necessary for Cd accumulation and tissue exhaustion, and the threshold level was not reached in a shorter time point (Chen et al. 2022; Nehzomi and Shirani 2025).

Exposure to clear air for 21 days significantly increased gut microbiota richness but not evenness in exposed mice (CdO/9w and CdO/CL/6w+21d). This effect was not observed in the pulmonary microbiome. This indicates that the clearance was more efficient in the gut microbiome. This is consistent with our analyses of Cd content in different organs, where the clearance period was found to be very effective for the intestine of exposed mice using the same inhalation protocol (Dumková et al. 2025). Moreover, the number of discriminately identified markers and dysregulated functions was higher in the colonic microbiome than in the pulmonary microbiome of both exposed groups (CdO/9w and CdO/CL/6w+21d). This illustrates that the effects of CdONP inhalation and clearance over a longer period were significantly more prominent in the gut microbiome than in the lung microbiome.

Notably, the abundance of *Ligilactobacillus* (formerly named *Lactobacillus*) was higher in the pulmonary microbiome of mice exposed to CdONPs for a longer period (CdO/9w, 15,23%) than in their control counterpart (KCd/9w, 0,62%) (Table S3). Similarly, *Lactobacillus* was the most influenced bacterial genus in the pulmonary microbiome of mice exposed to Cd aerosols (Tao et al. 2020). However, *Lactobacillus* decreased in the colonic microbiome of mice exposed to CdONPs for a longer period (CdO/9w, 7.43%) than in their control counterpart KCd/9w (16,26%) (Table S4). Consistent with these results, research studies showed that the exposure to Cd decreased the relative abundance of *Lactobacillus* in the gut microbiome of mice (Li et al. 2022), rats (Li et al. 2023), and hamsters (Tao et al. 2024).

Spearman’s correlation analysis uncovered a significantly positive association between “Electron transfer carriers” pathway and bacterial markers *Mediterraneibacter*, *Eubacterium*, *Lachnoclostridium*, and *Pseudoprevotella* in the lung microbiome of control mice (KCd/9w) in contrast to exposed mice (CdO/9w) (Fig. 7A). Microbial electron transfer carriers are important during metabolic reactions, particularly in metal respiring bacteria. They play a fundamental and indispensable role in cellular energy production (Kracke et al. 2015). CdONPs inhalation reduced the abundance of *Mediterraneibacter* in the lung microbiome, which resulted in the impairment of cellular function in energy production.

Lower abundance of *Roseburia* and *Mediterraneibacter* and higher abundance of *Kineothrix* were significantly correlated with “*N*-glycan biosynthesis,” “other ion-coupled transporters,” “peptidases,” “phosphonate and phosphinate metabolism,” and “primary immunodeficiency,” which were predicted in the pulmonary microbiome of the clearance group (CdO/CL/6w+21d) (Fig. 8B).

Reduced proportion of *Mediterraneibacter* and *Candidatus Athromitus* and increased proportion of *Duncaniella*, *Odoribacter* and *Pontibacter* were associated with higher predicted regulation of pathways in ion transporters and chelators, including “inorganic ion transport and metabolism,” “membrane and intracellular structural molecules,” “pores ion channels,” “protein folding and associated processing,” “protein digestion and absorption,” “lysosome,” “alpha-linolenic acid,” and “phosphonate and phosphinate metabolism,” “biosynthesis of siderophore group nonribosomal peptides” pathways, and lower predicted regulation of “Secretion system” pathway in the colonic microbiome of both exposed groups (CdO/9w, CdO/CL/6w+21d) (Fig. 8C and D). Heavy metals disrupt the microbial balance, potentially suppressing butyrate producers’ taxa like *Mediterraneibacter* through competitive exclusion by metal-resistant taxa (Bist and Choudhary 2022).

Previous studies have identified differential N-glycan patterns in lung adenocarcinoma tissue compared to healthy tissue, highlighting the relevance of N-glycan changes in disease progression (Lattová et al. 2020). Exposure to Cd can significantly impact the immune system, potentially contributing to immunodeficiency or exacerbating existing immune dysfunctions leading to immunosuppression, immune stimulation, hypersensitivity, and autoimmunity (Wang et al. 2021).

Biosynthesis of metal chelators and excretory compounds such as phosphonates, siderophores, and alpha-linolenic acid was predicted in the colonic microbiome of exposed mice (CdO/9w, CdO/CL/6w+21d). Phosphonates are known as strong metal chelating agents due to the presence of the phosphonic acid group, which can form a complex with metal ions, including cadmium, forming cadmium phosphonates (Hu et al. 2018; Kostelnik et al. 2021). Siderophores are produced by different bacterial species and possess ion‐chelating ability that can chelate primarily iron as well as several ions of heavy metals like Cd (Khan et al. 2020). The oral administration of the three fatty acids, including alpha-linolenic acid, significantly increased the fecal Cd levels, indicating the promotive effects of alpha-linolenic acid on Cd excretion (Fang et al. 2022). In agreement, our previous experiments revealed a higher accumulation of Cd in the intestinal content than in surrounding intestinal tissues of the mice after exposure to CdONPs (Dumková et al. 2025) while using the same experimental approach used in this study.

The gut microbiota acts as a first line of defense against the toxic effects of heavy metals, and there is a bidirectional relationship between Cd exposure and the gut microbiota. Cd inhalation disrupts homeostasis, taxonomic composition, and metabolic profile of the gut microbiota, and in turn, the gut microbiome regulates the absorption, bioavailability, and excretion of Cd (Duan et al. 2020; Santiago et al. 2024). Cd exposure alters the composition and structure of the gut microbiota, often leading to a state of “dysbiosis”. Dysbiosis may result in an impaired metabolic functional profile of the gut microbiota (Tao et al. 2020; Yang et al. 2021; Liu et al. 2023) and induce alterations in gut morphology and the integrity of the intestinal barrier (He et al. 2020b; Liu et al. 2020).

The capacity of some selected gut bacteria was explored for their ability to remove Cd in vitro while using the strategy of “intestinal bioremediation” (George et al. 2021). An in vitro study determined that the gut microbial community induced a significant decrease in the intestinal permeability of Cd, thereby mitigating Cd toxicity (Bolan et al. 2021). However, the mechanism of bacteria in removing or reducing Cd from the intestine was not elucidated. Microorganisms can reduce the bioavailability of Cd through mechanisms like biosorption, bioaccumulation, and detoxification (Ayangbenro and Babalola 2017; Dave et al. 2020). A recent study has reported the bioaccumulation of polyfluoroalkyl substances by multiple human gut bacterial strains, showing the ability of human gut bacteria to uptake pollutants (Lindell et al. 2025). Bioaccumulation refers to the transport of heavy metal ions across the cell membrane and their accumulation inside the microbial cell using inner membrane transporters, channels and carriers (Diep et al. 2018). When the role of the gut microbiota in the bioaccumulation and retention of Cd in primary organs of germ-free and SPF mice subjected to oral exposure to Cd was evaluated (Breton et al. 2013), significant differences in basal gene expression related to divalent metal transporters were found between control and germ-free mice (Breton et al. 2013). Moreover, “ion-coupled transporters,” “membrane and intracellular structural molecules,” “pores ion channels,” “protein folding and associated processing,” “protein digestion and absorption,” and “lysosome,” play a critical role in moving ions across membranes (Nikaido and Saier 1992; Chen et al. 2022; Özkan et al. 2024). Genes related to those important transporters were significantly positively correlated with the abundance of *Duncaniella* in the colonic microbiome of both the exposure (CdO/9w) and clearance (CdO/CL/6w+21d) groups. Network differential analysis determined that *Duncaniella* positively correlated with *Ligilactobacillus* in the colonic microbiome of exposed mice (CdO/9w). Similar to our results, *Duncaniella* was previously found to exhibit significant variations in the gut microbiota of *Cricetulus longicaudatus* due to Cd exposure (Tao et al. 2024). These analyses identified intestinal *Duncaniella* as a contributor to the protection of *Dusp*6-deficient mice from DSS-induced colitis (Chang et al. 2021). It was suggested that administration of *Lactobacillus plantarum* may increase Cd excretion by regulating the enterohepatic circulation of bile acids in a gut microbiota-dependant manner, resulting in a marked reduction in the content of Cd in tissue and alleviating Cd poisoning (Zhai et al. 2019a).

Previous studies have demonstrated significant enhancement of the relative abundance of *Odoribacter* in the intestinal microbiota of mice after Cd exposure, similar to our results (Zhai et al. 2017a). These findings indicate that *Odoribacter splanchnicus* exerts an immunoregulatory effect, which can mitigate gut inflammation conditions like inflammatory bowel disease (Hiippala et al. 2020). Nonetheless, no correlation has been established between *Pontibacter* and Cd exposure in earlier studies.

The human gut bacterium *Enterococcus faecalis* strain ATCC19433 was found to have the ability to remove Cd from an aqueous solution (Zheng et al. 2024). Characterization analyses revealed notable changes in the morphology of bacterial cell walls. Results revealed that Cd removal mechanisms by this bacterial strain were predominantly dependent on biosorption on the cell surface and intracellular bioaccumulation. Bacterial surfaces of this strain containing alkyl, amide II, and phosphate groups were able to bind to Cd. Moreover, genes encoding metal ion transport proteins that may drive the translocation of Cd into the intracellular region were found in its genome (Zheng et al. 2024). Interestingly, our previous analyses uncovered that Cd concentration in the intestines was very low after CdONPs inhalation (Dumková et al. 2025). Altogether, these results suggest that Cd exposure conducts the alterations of the gut microbiota functions and may cause the removal of Cd from the intestine by two potential ways: (i) increase in biosynthesis of Cd chelators within the intestinal lumen may support the subsequent elimination of Cd from the body through fecal excretion and (ii) increase in proportion level of bacterial membrane transporters leading to the accumulation of Cd inside the bacterial cell and the decrease of Cd concentration in the intestinal tissue. Moreover, previous studies determined that the oral administration of probiotics protects the intestinal barrier by reducing the intestinal absorption of Cd in mice and increasing Cd excretion (Zhai et al. 2016, 2017b; Zhu et al. 2021). Furthermore, supplementation with galactooligosaccharides could work as a potential protective prebiotic against Pb toxicity through modulation of the gut microbiota by enhancing the abundance of intestinal bacteria with good Pb-binding ability (Zhai et al. 2019b). Therefore, probiotics, prebiotics or synbiotic supplementation are regarded as promising strategies for the future to alleviate the toxicological effects of Cd.

Given the limitation of the 16S-based functional prediction, future study incorporating metagenomic and metatranscriptomic sequencing will be necessary to directly validate the functional hypothesis proposed here. A further limitation of this study is that no multiple-testing correction was applied to the *P*-values in the functional prediction analysis. These results should therefore be interpreted with caution because *P*-values may include inflated false-positive rates. The relatively small sample size within each group (*n* = 5) used in this study represents another limitation of this study, which may reduce the statistical stability of the analyses. In the correlation analysis, interpretation was based on Spearman’s correlation coefficients and raw *p*‑values. FDR correction assumes independence among tests, but microbial abundances are compositional and strongly correlated, making Benjamini–Hochberg adjustment overly conservative in small pre‑selected feature sets. As shown in Supplementary Table S8, BH-correction markedly reduced significance, often eliminating associations that were biologically plausible. For these reasons, raw *p*‑values were interpreted cautiously, while corrected values were reported for completeness. Another limitation of this study is the absence of baseline microbiome profiling prior to CdONPs exposure, which prevents direct assessment of inter-individual variability and its potential influence on the observed microbial responses. Although mice originated from different breeding cages, they were mixed during a one-week acclimatization period and subsequently randomly assigned to experimental groups, and all animals could be co-housed under identical environmental conditions (reflecting inhalation conditions). However, our design was intended to minimize cage- and origin-related effects and to distribute any pre-existing microbiome differences across groups. Such co-housing strategies are commonly used to reduce inter-cage variability; however, baseline differences in microbiome composition cannot be entirely excluded. Consequently, it remains possible that part of the observed variation in microbiome response to Cd exposure reflects pre-existing microbial differences or host-microbiome interactions, rather than exposure effects alone. Future studies incorporating longitudinal sampling of the same individuals before, during, and after exposure, or using approaches such as microbiome normalization strategies, would help to better disentangle baseline variability from treatment-induced changes and strengthen causal interpretation.

## Conclusion

The impact of CdONPs exposure was more pronounced in the gut microbiome than in the pulmonary microbiome. CdONPs exposure appears to modulate gut microbial eubiosis in a manner that may enhance host resilience to Cd‑induced toxicity. Further studies are needed to explore the possible therapeutic strategies using beneficial microorganisms with metal-absorption and metal-binding capabilities as a potential probiotic to be supplemented to mitigate heavy metal toxicity.

## Supplementary Information

Below is the link to the electronic supplementary material.

ESM 1 (PDF 8.72 MB)

ESM 2 (XLSX 12.0 KB)

ESM 3 (XLSX 12.8 KB)

ESM 4 (XLSX 35.6 KB)

ESM 5 (XLSX 24.3 KB)

ESM 6 (XLSX 19.9 KB)

ESM 7 (XLSX 19.0 KB)

ESM 8 (XLSX 24.3 KB)

ESM 9 (XLSX 48.3)

## Data Availability

The nucleotide sequences have been submitted in the NCBI database in Sequence Read Archive (SRA) SUB15400658 under BioProject ID: PRJNA1279427.

## Abbreviations

ASV: Amplicon sequence variant
Cd: Cadmium
CdONPs: Inhalable CdO nanoparticles
KEGG: Kyoto Encyclopedia of Genes and Genomes
PCoA: Principal coordinate analysis
PICRUSt: Phylogenetic investigation of communities by reconstruction of unobserved states
SCFA: Short-chain fatty acids
SPF: Specific pathogen-free
WHO: World Health Organization
