# Supplementary material for: Inhalation of cadmium oxide nanoparticles alters intestinal and pulmonary microbiomes in mice
Source: Appl Microbiol Biotechnol. 2026 Jul 28;110:232. doi: 10.1007/s00253-026-13970-3 (PMC13415660; doi:10.1007/s00253-026-13970-3)
Supplement: Supplementary file 1 [file 253_2026_13970_MOESM1_ESM.pdf]

Applied Microbiology and Biotechnology

Inhalation of cadmium oxide nanoparticles alters intestinal and pulmonary microbiomes in mice

Chahrazed Mekadim[a], Daniela Kristeková[b,c], Pavel Mikuška[d], Marcela Buchtová[b,c], Jakub Mrázek[a]

[a] Laboratory of anaerobic microbiology, Institute of Animal Physiology and Genetics, Czech Academy of Sciences, Prague 1083, Czech Republic.

[b]Laboratory of Molecular Morphogenesis, Institute of Animal Physiology and Genetics, Czech Academy of Sciences, Brno 602 00, Czech Republic.

[c]Department of Experimental Biology, Faculty of Science, Masaryk University, Brno 625 00, Czech Republic

[d] Department of Environmental Analytical Chemistry, Institute of Analytical Chemistry, Czech Academy of Sciences, Brno 602 00, Czech Republic

Correspondence: mekadim@iapg.cas.cz

Address: Laboratory of Anaerobic Microbiology, Institute of Animal Physiology and Genetics of the Czech Academy of Sciences, Videnska 1083, 142 00, Prague, Czech Republic

Supplementary figures

Figure S1 Effect of CdONPs exposure on the pulmonary and gut microbiota composition. Relative abundance of bacterial communities at phylum level in microbiomes of lungs (A) and colon (B) of exposed mice for a shorter time (6w+1d period) and longer time (6w+21d period).

(size sample for each group: n=5).

Figure S2 Co-occurrence Network Analysis in the pulmonary and gut microbiome of mice exposed to CdONPs for a shorter period. Each node represents a bacterial genus. The right panel shows the colors of the edges between taxa (the thicker the line, the stronger the association): (+) indicates a positive association, (-) indicates an anti-correlation and (0) indicates an absent association between taxa in a specific group based on Spearman's correlation coefficient in the microbiome of lungs (A, B) and colon (C, D) of control and CdONPs exposed mice for a shorter period (KCd/6w+1d, CdO/6w+1d, CdO/CL/6w+1d). (size sample for each group: n=5).

Figure S3 Co-occurrence Network Analysis in the pulmonary and gut microbiome of mice exposed to CdONPs for a longer period. Each node represents a bacterial genus. The right panel shows the colors of the edges between taxa (the thicker the line, the stronger the association): (+) indicates a positive association, (-) indicates an anti-correlation and (0) indicates an absent association between taxa in a specific group based on Spearman's correlation coefficient in the microbiome of lungs (A, B) and colon (C, D) of control and CdONPs exposed mice for longer period (KCd/9w, CdO/9w, CdO/CL/6w+21d). (size sample for each group: n=5).

Figure S4 Effect of CdONPs exposure on the functional pathways of the pulmonary and gut microbiota of mice exposed for a shorter period. Predicted functional KEGG pathways at level 3 in the microbiome of lungs (A) and colon (B) of mice exposed to CdONPs for a shorter

period (CdO/6w+1d) in contrast to their control counterpart (KCd/6w+1d). $P < 0.05$ was considered statistically significant. (size sample for each group: n=5).

Figure S5 Effect of CdONPs exposure on the functional pathways of the pulmonary and gut microbiota of mice with shorter clearance period. Predicted functional KEGG pathways at level 3 in the microbiome of lungs (A) and colon (B) of mice exposed to CdONPs for 6 weeks and then exposed to clean air for 1 day (CdO/CL/6w+1d) in contrast to their control counterpart (KCd/6w+1d). (size sample for each group: n=5).

Figure S6 Effect of CdONPs exposure on the functional pathways of the pulmonary and gut microbiota of mice exposed for a longer period. Predicted functional KEGG pathways at level 3 in the microbiome of lungs (A) and colon (B) of mice exposed to CdONPs for a longer period (CdO/9w) in contrast to their control counterpart (KCd/9w). $P < 0.05$ was considered statistically significant. (size sample for each group: n=5).

Figure S7 Effect of Cd exposure on the functional pathways of the pulmonary and gut microbiota of mice with longer clearance period. Predicted functional KEGG pathways at level 3 in the microbiome of lungs (A) and colon (B) of mice exposed to CdONPs for 6 weeks and then exposed to clean air for 21 days (CdO/CL/6w+21d) in contrast to their control counterpart (KCd/9w). $P < 0.05$ was considered statistically significant. (size sample for each group: n=5).

Figure S1

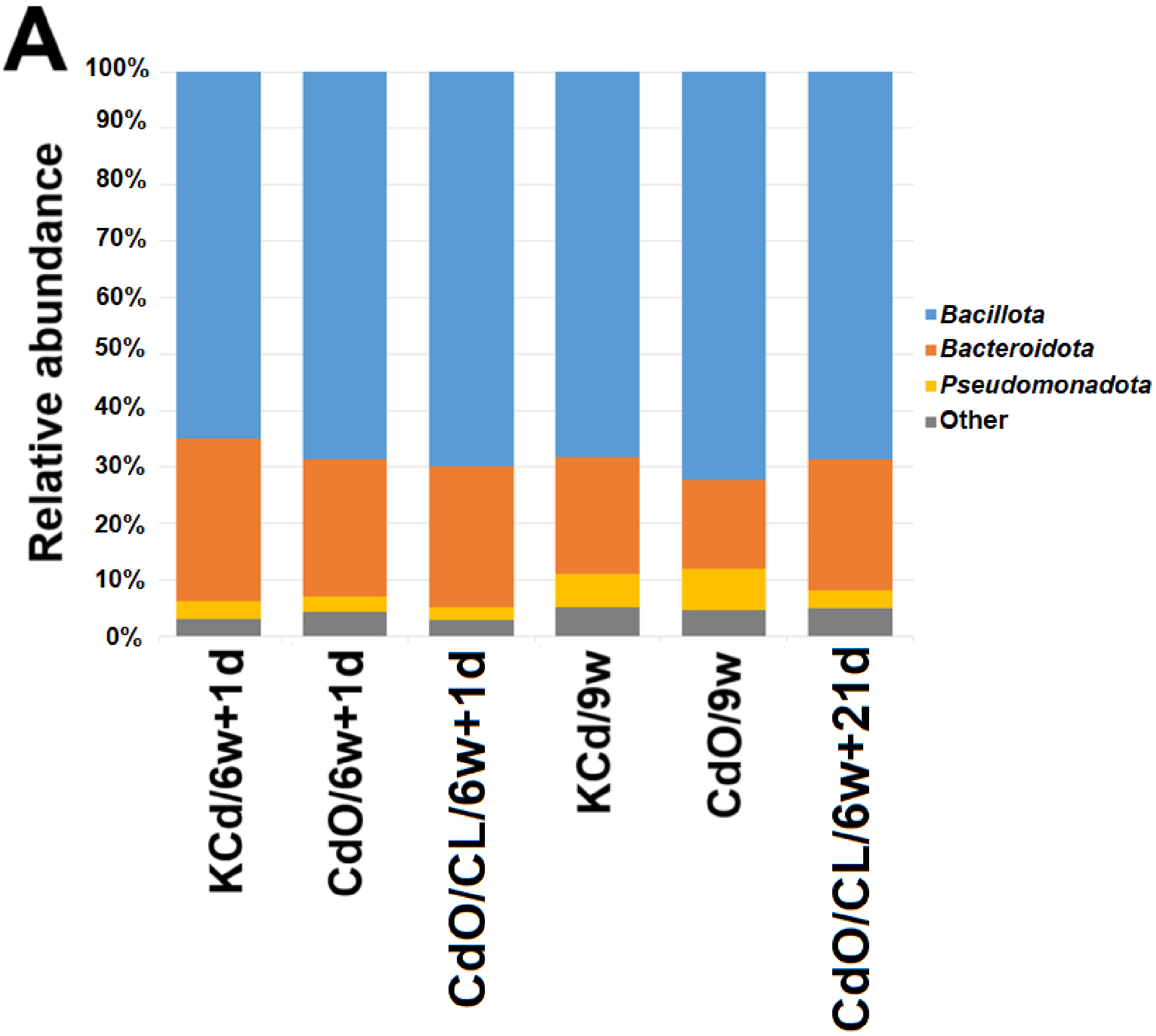
A
Relative abundance
100%
90%
80%
70%
60%
50%
40%
30%
20%
10%
0%
KCd/6w+1d
CdO/6w+1d
CdO/CL/6w+1d
KCd/9w
CdO/9w
CdO/CL/6w+21d
Bacillota
Bacteroidota
Pseudomonadota
Other

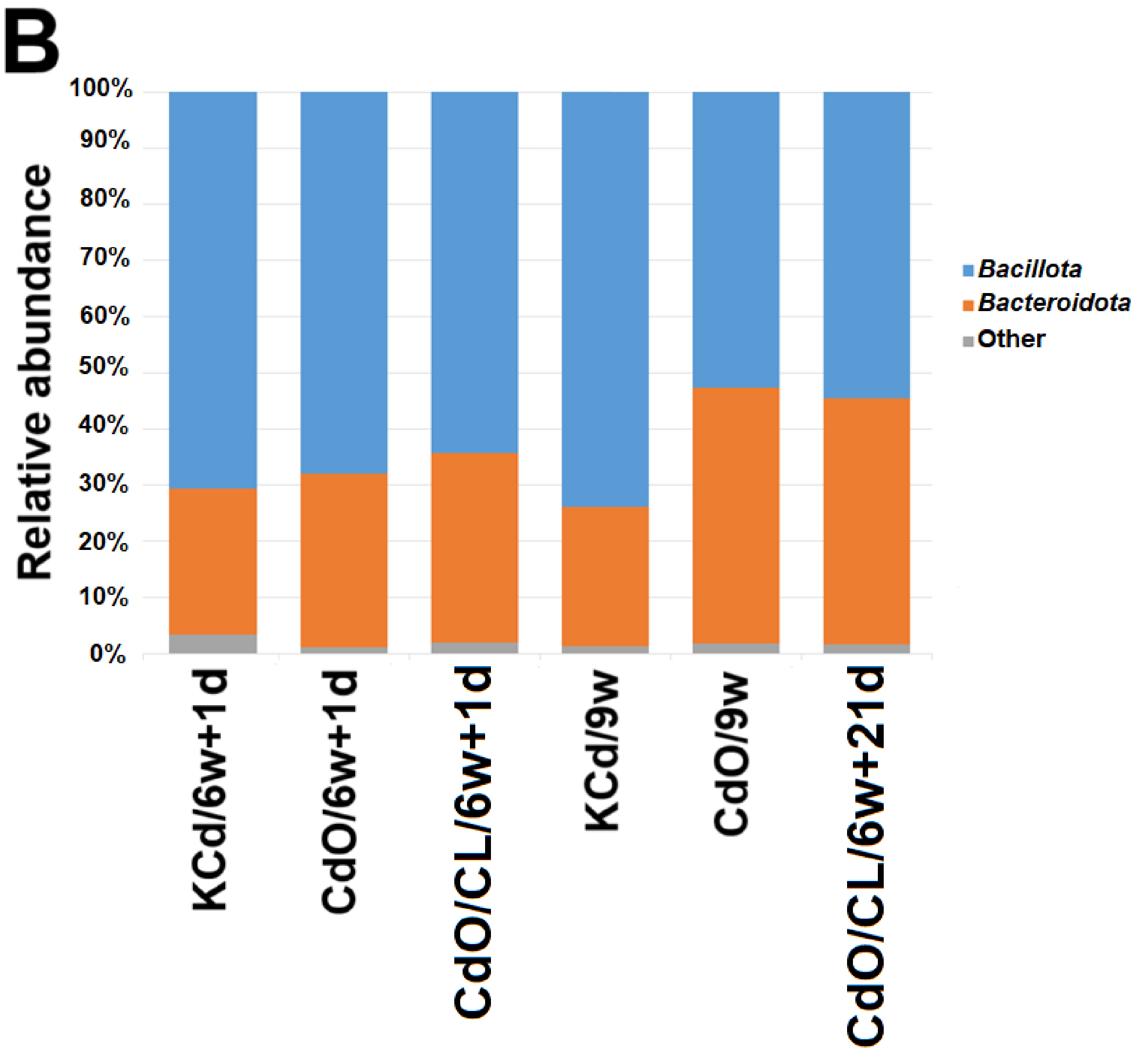
B
Relative abundance
100%
90%
80%
70%
60%
50%
40%
30%
20%
10%
0%
KCd/6w+1d
CdO/6w+1d
CdO/CL/6w+1d
KCd/9w
CdO/9w
CdO/CL/6w+21d
Bacillota
Bacteroidota
Other

Figure S2

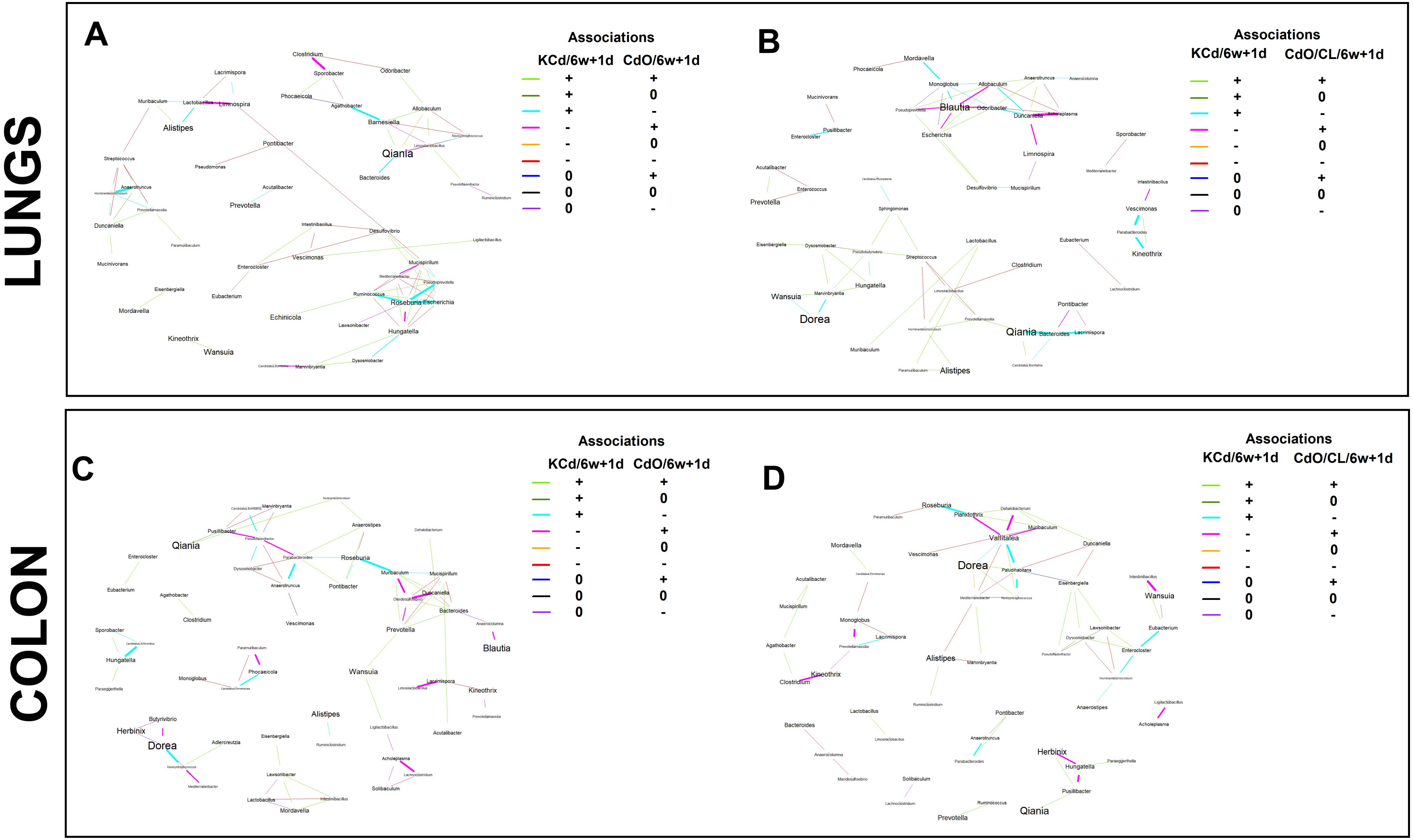
LUNGS
COLON
A
Associations
KCd/6w+1d CdO/6w+1d
+ +
+ 0
+ -
- +
- 0
- -
0 +
0 0
0 -
Clostridium
Sporobacter
Odoribacter
Lacrimispora
Muribaculum
Lactobacillus
Limnospira
Phocaeicola
Agathobacter
Allobaculum
Alistipes
Barnesiella
Pontibacter
Qiania
Streptococcus
Pseudomonas
Bacteroides
Anaerotruncus
Acutalibacter
Prevotellamassilia
Prevotella
Duncaniella
Intestinibacillus
Desulfovibrio
Paramuribaculum
Vescimonas
Mucinivorans
Enterocloster
Mucispirillum
Eisenbergiella
Eubacterium
Ruminococcus
Roseburia
Escherichia
Mordavella
Echinicola
Lawsonibacter
Hungatella
Kineothrix
Wansuia
Dysosmobacter
Marvinbryantia
B
Associations
KCd/6w+1d CdO/CL/6w+1d
+ +
+ 0
+ -
- +
- 0
- -
0 +
0 0
0 -
Mordavella
Phocaeicola
Monoglobus
Allobaculum
Anaerotruncus
Mucinivorans
Blautia
Odoribacter
Duncaniella
Acholeplasma
Pusillibacter
Enterocloster
Escherichia
Limnospira
Sporobacter
Acutalibacter
Enterococcus
Desulfovibrio
Mucispirillum
Prevotella
Sphingomonas
Vescimonas
Eisenbergiella
Dysosmobacter
Streptococcus
Lactobacillus
Eubacterium
Kineothrix
Clostridium
Hungatella
Wansuia
Dorea
Pontibacter
Qiania
Bacteroides
Lacrimispora
Muribaculum
Alistipes
C
Associations
KCd/6w+1d CdO/6w+1d
+ +
+ 0
+ -
- +
- 0
- -
0 +
0 0
0 -
Marvinbryantia
Anaerostipes
Pusillibacter
Qiania
Enterocloster
Parabacteroides
Roseburia
Muribaculum
Mucispirillum
Eubacterium
Agathobacter
Anaerotruncus
Pontibacter
Duncaniella
Bacteroides
Clostridium
Vescimonas
Prevotella
Sporobacter
Blautia
Hungatella
Phocaeicola
Monoglobus
Wansuia
Lacrimispora
Kineothrix
Alistipes
Butyrivibrio
Herbinix
Dorea
Adlercreutzia
Eisenbergiella
Acutalibacter
Acholeplasma
Lawsonibacter
Solibaculum
Lactobacillus
Intestinibacillus
Mordavella
D
Associations
KCd/6w+1d CdO/CL/6w+1d
+ +
+ 0
+ -
- +
- 0
- -
0 +
0 0
0 -
Roseburia
Planktothrix
Muribaculum
Vallitalea
Duncaniella
Mordavella
Vescimonas
Dorea
Paludihabitans
Acutalibacter
Eisenbergiella
Wansuia
Mucispirillum
Monoglobus
Lacrimispora
Lawsonibacter
Eubacterium
Agathobacter
Alistipes
Enterocloster
Kineothrix
Clostridium
Marvinbryantia
Anaerostipes
Acholeplasma
Lactobacillus
Pontibacter
Bacteroides
Anaerotruncus
Herbinix
Hungatella
Solibaculum
Pusillibacter
Qiania
Prevotella

Figure S3

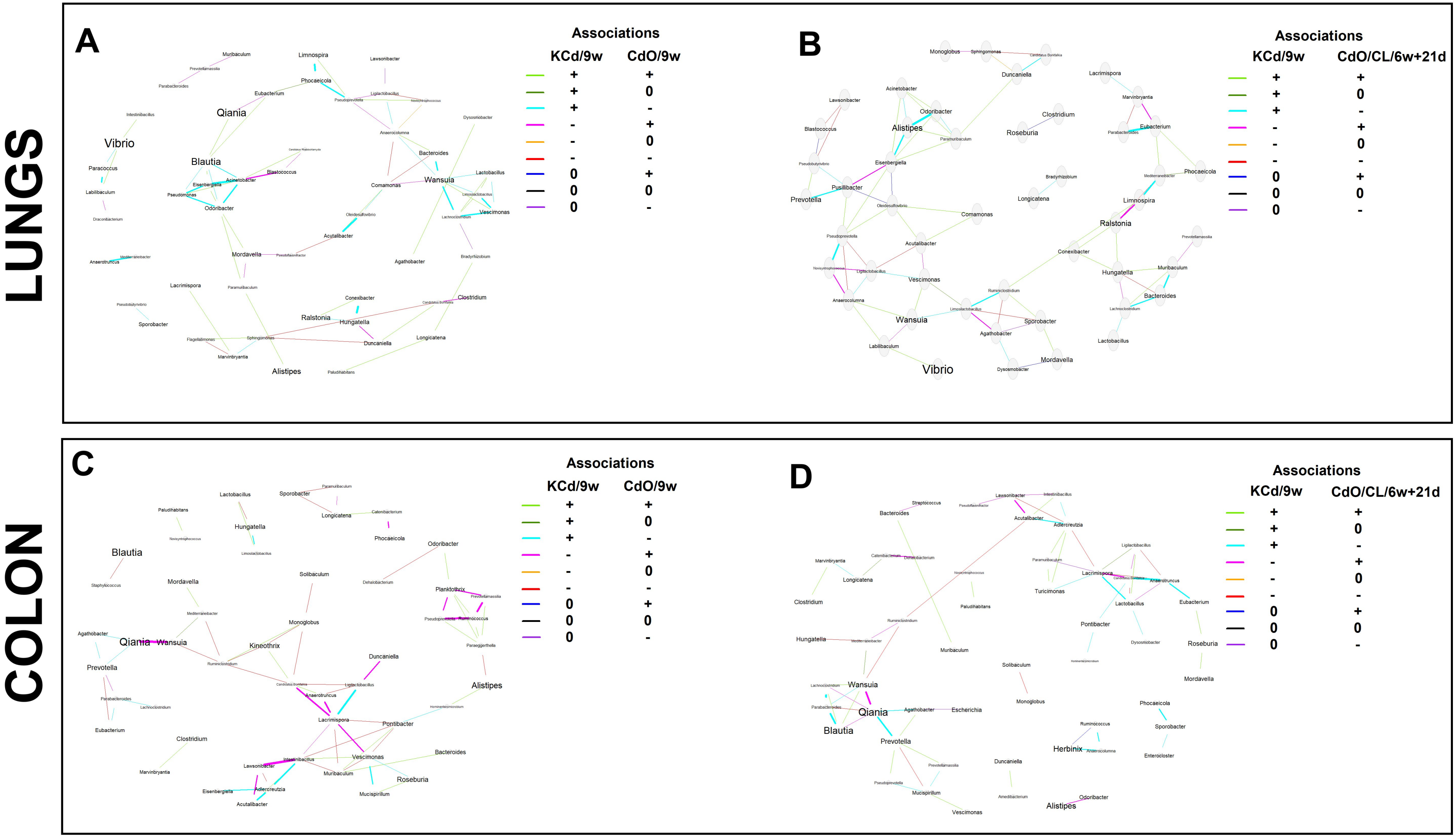

LUNGS
COLON
A
Associations
KCd/9w CdO/9w
+ +
+ 0
+ -
- +
- 0
- -
0 +
0 0
0 -
B
Associations
KCd/9w CdO/CL/6w+21d
+ +
+ 0
+ -
- +
- 0
- -
0 +
0 0
0 -
C
Associations
KCd/9w CdO/9w
+ +
+ 0
+ -
- +
- 0
- -
0 +
0 0
0 -
D
Associations
KCd/9w CdO/CL/6w+21d
+ +
+ 0
+ -
- +
- 0
- -
0 +
0 0
0 -
Vibrio
Qiania
Blautia
Wansuia
Alistipes
Prevotella
Ralstonia
Hungatella
Herbinix

Figure S4

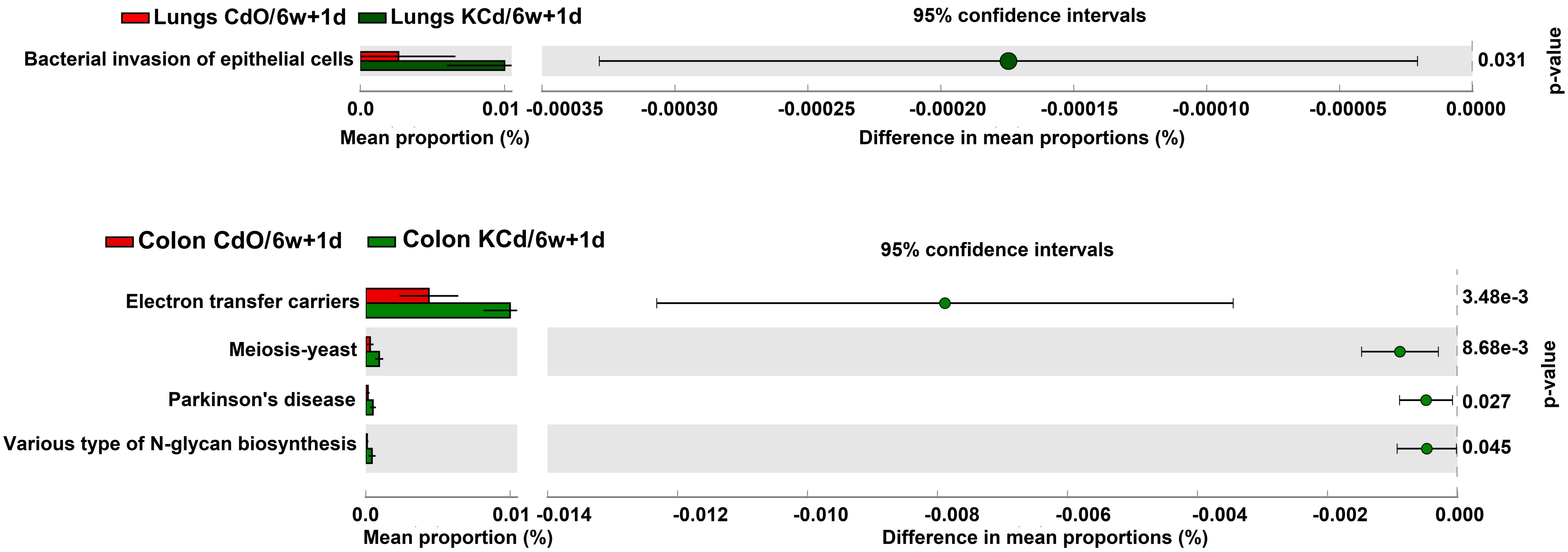

Lungs CdO/6w+1d
Lungs KCd/6w+1d
95% confidence intervals
Bacterial invasion of epithelial cells
0.031
p-value
0.0
0.01
Mean proportion (%)
-0.00035
-0.00030
-0.00025
-0.00020
-0.00015
-0.00010
-0.00005
0.0000
Difference in mean proportions (%)
Colon CdO/6w+1d
Colon KCd/6w+1d
95% confidence intervals
Electron transfer carriers
Meiosis-yeast
Parkinson's disease
Various type of N-glycan biosynthesis
3.48e-3
8.68e-3
0.027
0.045
p-value
0.0
0.01
Mean proportion (%)
-0.014
-0.012
-0.010
-0.008
-0.006
-0.004
-0.002
0.000
Difference in mean proportions (%)

Figure S5

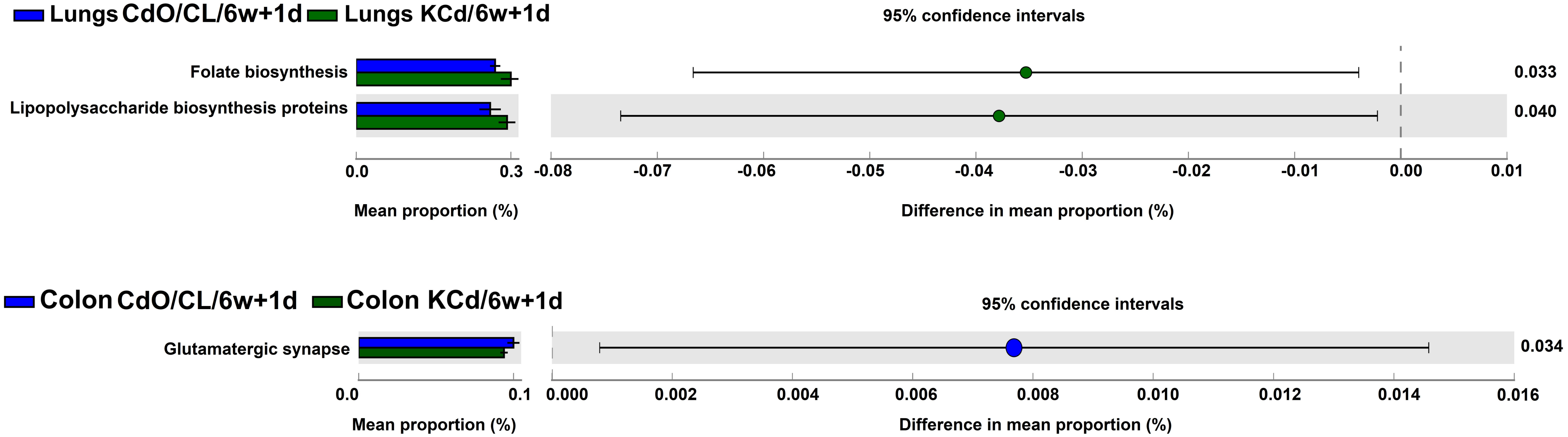

Lungs CdO/CL/6w+1d
Lungs KCd/6w+1d
95% confidence intervals
Folate biosynthesis
Lipopolysaccharide biosynthesis proteins
0.033
0.040
0.0
0.3
-0.08
-0.07
-0.06
-0.05
-0.04
-0.03
-0.02
-0.01
0.00
0.01
Mean proportion (%)
Difference in mean proportion (%)
Colon CdO/CL/6w+1d
Colon KCd/6w+1d
95% confidence intervals
Glutamatergic synapse
0.034
0.0
0.1
0.000
0.002
0.004
0.006
0.008
0.010
0.012
0.014
0.016
Mean proportion (%)
Difference in mean proportion (%)

Figure S6

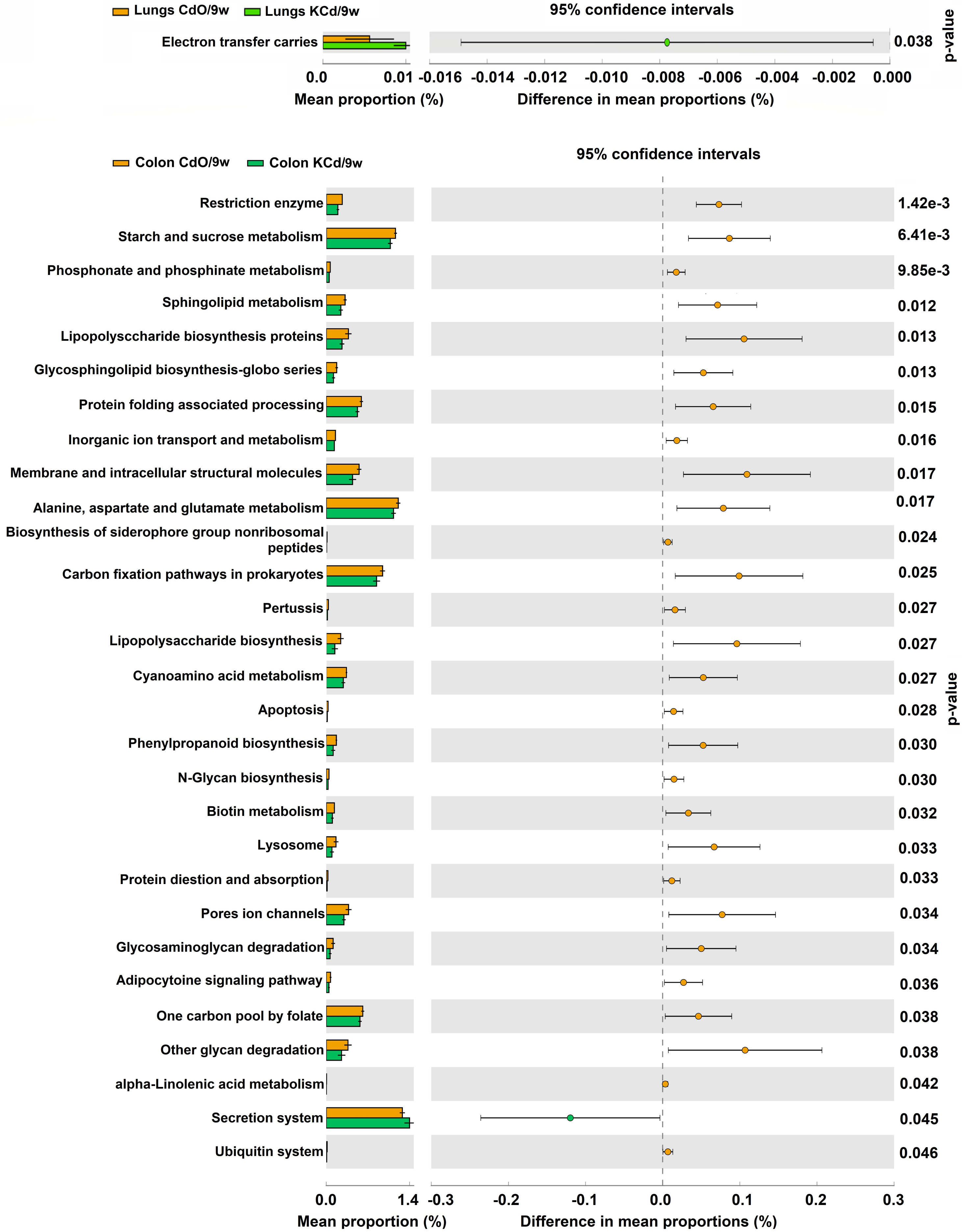
Lungs CdO/9w
Lungs KCd/9w
95% confidence intervals
Electron transfer carries
0.038
p-value
0.0
0.01
-0.016
-0.014
-0.012
-0.010
-0.008
-0.006
-0.004
-0.002
0.000
Mean proportion (%)
Difference in mean proportions (%)
Colon CdO/9w
Colon KCd/9w
95% confidence intervals
Restriction enzyme
1.42e-3
Starch and sucrose metabolism
6.41e-3
Phosphonate and phosphinate metabolism
9.85e-3
Sphingolipid metabolism
0.012
Lipopolyscchride biosynthesis proteins
0.013
Glycosphingolipid biosynthesis-globo series
0.013
Protein folding associated processing
0.015
Inorganic ion transport and metabolism
0.016
Membrane and intracellular structural molecules
0.017
Alanine, aspartate and glutamate metabolism
0.017
Biosynthesis of siderophore group nonribosomal peptides
0.024
Carbon fixation pathways in prokaryotes
0.025
Pertussis
0.027
Lipopolysaccharide biosynthesis
0.027
Cyanoamino acid metabolism
0.027
Apoptosis
0.028
Phenylpropanoid biosynthesis
0.030
N-Glycan biosynthesis
0.030
Biotin metabolism
0.032
Lysosome
0.033
Protein diestion and absorption
0.033
Pores ion channels
0.034
Glycosaminoglycan degradation
0.034
Adipocytoine signaling pathway
0.036
One carbon pool by folate
0.038
Other glycan degradation
0.038
alpha-Linolenic acid metabolism
0.042
Secretion system
0.045
Ubiquitin system
0.046
p-value
0.0
1.4
-0.3
-0.2
-0.1
0.0
0.1
0.2
0.3
Mean proportion (%)
Difference in mean proportions (%)

Figure S7

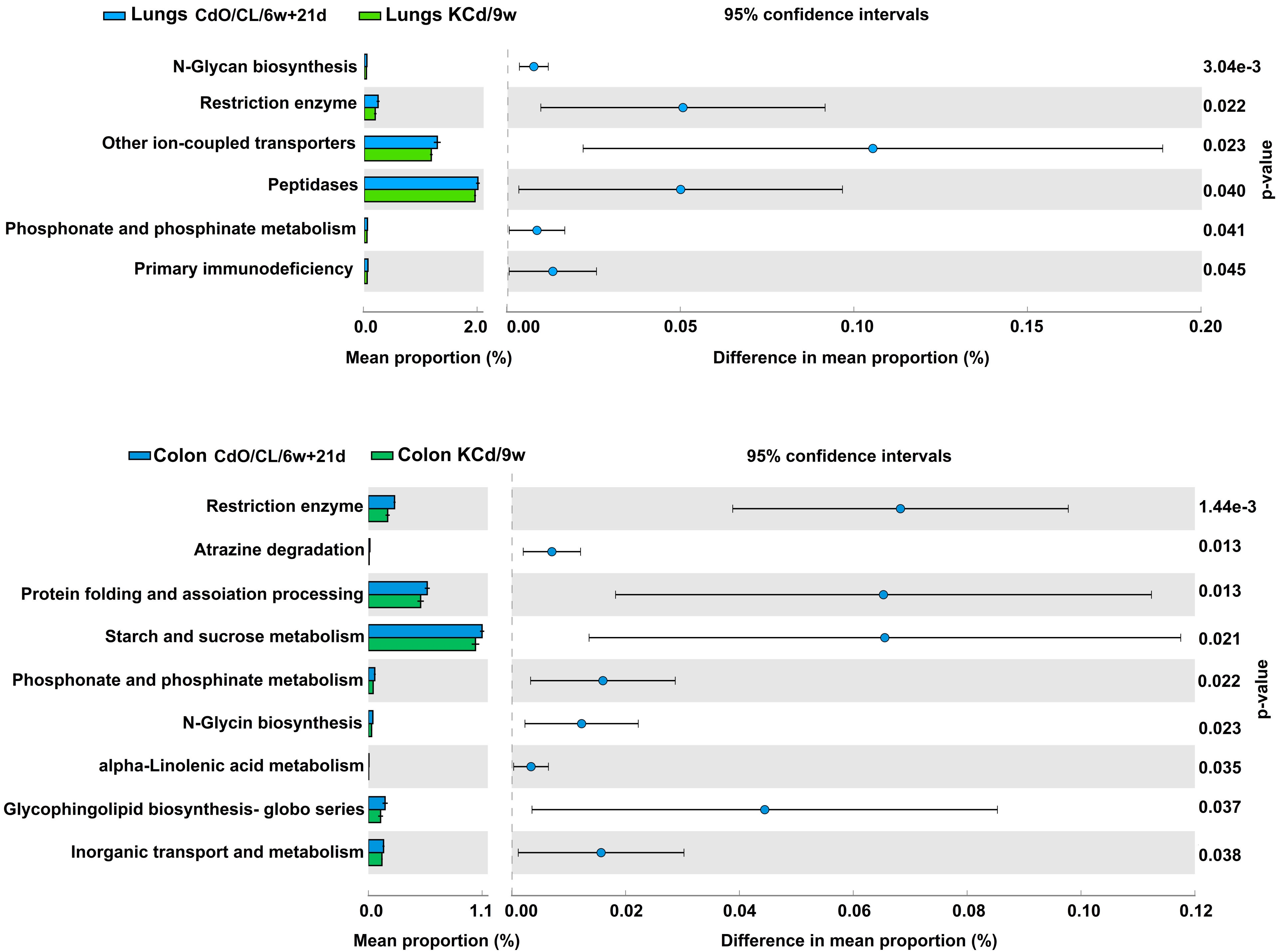

Lungs CdO/CL/6w+21d
Lungs KCd/9w
95% confidence intervals
N-Glycan biosynthesis
Restriction enzyme
Other ion-coupled transporters
Peptidases
Phosphonate and phosphinate metabolism
Primary immunodeficiency
3.04e-3
0.022
0.023
0.040
0.041
0.045
p-value
0.0
2.0
0.00
0.05
0.10
0.15
0.20
Mean proportion (%)
Difference in mean proportion (%)
Colon CdO/CL/6w+21d
Colon KCd/9w
95% confidence intervals
Restriction enzyme
Atrazine degradation
Protein folding and assoiation processing
Starch and sucrose metabolism
Phosphonate and phosphinate metabolism
N-Glycin biosynthesis
alpha-Linolenic acid metabolism
Glycophingolipid biosynthesis- globo series
Inorganic transport and metabolism
1.44e-3
0.013
0.013
0.021
0.022
0.023
0.035
0.037
0.038
p-value
0.0
1.1
0.00
0.02
0.04
0.06
0.08
0.10
0.12
Mean proportion (%)
Difference in mean proportion (%)
