# Supplementary material for: Inhalation of cadmium oxide nanoparticles alters intestinal and pulmonary microbiomes in mice
Source: Appl Microbiol Biotechnol. 2026 Jul 28;110(1):232. doi: 10.1007/s00253-026-13970-3 (PMC13415660; doi:10.1007/s00253-026-13970-3)
Supplement: Supplementary file 1 — (PDF 8.72 MB) [file 253_2026_13970_MOESM1_ESM.pdf]

Applied Microbiology and Biotechnology

Inhalation of cadmium oxide nanoparticles alters intestinal and pulmonary microbiomes in mice

Chahrazed Mekadim[a], Daniela Kristeková[b,c], Pavel Mikuška[d], Marcela Buchtová[b,c], Jakub Mrázek[a]

[a] Laboratory of anaerobic microbiology, Institute of Animal Physiology and Genetics, Czech Academy of Sciences, Prague 1083, Czech Republic.

[b]Laboratory of Molecular Morphogenesis, Institute of Animal Physiology and Genetics, Czech Academy of Sciences, Brno 602 00, Czech Republic.

[c]Department of Experimental Biology, Faculty of Science, Masaryk University, Brno 625 00, Czech Republic

[d] Department of Environmental Analytical Chemistry, Institute of Analytical Chemistry, Czech Academy of Sciences, Brno 602 00, Czech Republic

Correspondence: mekadim@iapg.cas.cz

Address: Laboratory of Anaerobic Microbiology, Institute of Animal Physiology and Genetics of the Czech Academy of Sciences, Videnska 1083, 142 00, Prague, Czech Republic

Supplementary figures

Figure S1 Effect of CdONPs exposure on the pulmonary and gut microbiota composition. Relative abundance of bacterial communities at phylum level in microbiomes of lungs (A) and colon (B) of exposed mice for a shorter time (6w+1d period) and longer time (6w+21d period).

(size sample for each group: n=5).

Figure S2 Co-occurrence Network Analysis in the pulmonary and gut microbiome of mice exposed to CdONPs for a shorter period. Each node represents a bacterial genus. The right panel shows the colors of the edges between taxa (the thicker the line, the stronger the association): (+) indicates a positive association, (-) indicates an anti-correlation and (0) indicates an absent association between taxa in a specific group based on Spearman's correlation coefficient in the microbiome of lungs (A, B) and colon (C, D) of control and CdONPs exposed mice for a shorter period (KCd/6w+1d, CdO/6w+1d, CdO/CL/6w+1d). (size sample for each group: n=5).

Figure S3 Co-occurrence Network Analysis in the pulmonary and gut microbiome of mice exposed to CdONPs for a longer period. Each node represents a bacterial genus. The right panel shows the colors of the edges between taxa (the thicker the line, the stronger the association): (+) indicates a positive association, (-) indicates an anti-correlation and (0) indicates an absent association between taxa in a specific group based on Spearman's correlation coefficient in the microbiome of lungs (A, B) and colon (C, D) of control and CdONPs exposed mice for longer period (KCd/9w, CdO/9w, CdO/CL/6w+21d). (size sample for each group: n=5).

Figure S4 Effect of CdONPs exposure on the functional pathways of the pulmonary and gut microbiota of mice exposed for a shorter period. Predicted functional KEGG pathways at level 3 in the microbiome of lungs (A) and colon (B) of mice exposed to CdONPs for a shorter

period (CdO/6w+1d) in contrast to their control counterpart (KCd/6w+1d). $P < 0.05$ was considered statistically significant. (size sample for each group: n=5).

Figure S5 Effect of CdONPs exposure on the functional pathways of the pulmonary and gut microbiota of mice with shorter clearance period. Predicted functional KEGG pathways at level 3 in the microbiome of lungs (A) and colon (B) of mice exposed to CdONPs for 6 weeks and then exposed to clean air for 1 day (CdO/CL/6w+1d) in contrast to their control counterpart (KCd/6w+1d). (size sample for each group: n=5).

Figure S6 Effect of CdONPs exposure on the functional pathways of the pulmonary and gut microbiota of mice exposed for a longer period. Predicted functional KEGG pathways at level 3 in the microbiome of lungs (A) and colon (B) of mice exposed to CdONPs for a longer period (CdO/9w) in contrast to their control counterpart (KCd/9w). $P < 0.05$ was considered statistically significant. (size sample for each group: n=5).

Figure S7 Effect of Cd exposure on the functional pathways of the pulmonary and gut microbiota of mice with longer clearance period. Predicted functional KEGG pathways at level 3 in the microbiome of lungs (A) and colon (B) of mice exposed to CdONPs for 6 weeks and then exposed to clean air for 21 days (CdO/CL/6w+21d) in contrast to their control counterpart (KCd/9w). $P < 0.05$ was considered statistically significant. (size sample for each group: n=5).

Figure S1

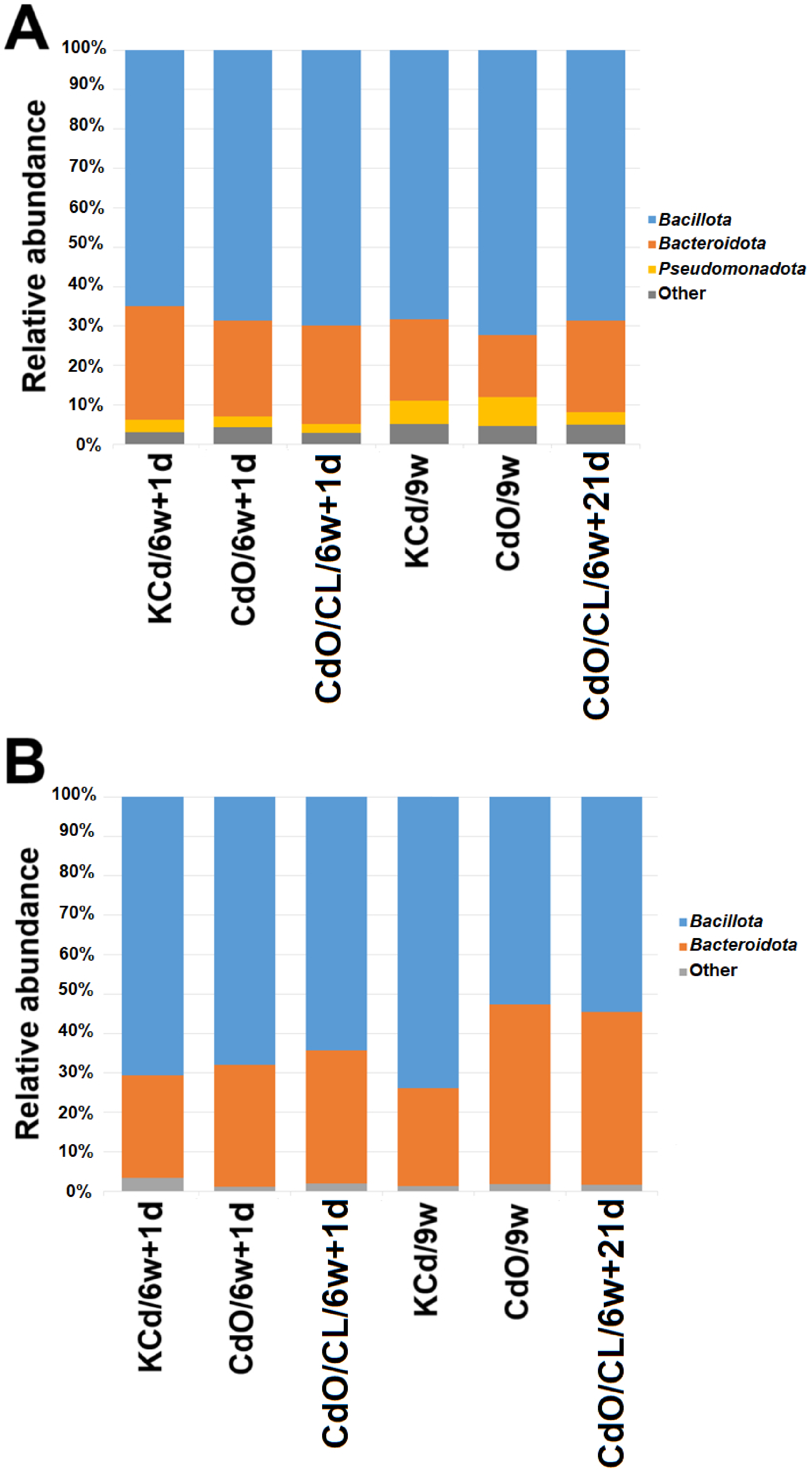
A
Relative abundance
100%
90%
80%
70%
60%
50%
40%
30%
20%
10%
0%
KCd/6w+1d
CdO/6w+1d
CdO/CL/6w+1d
KCd/9w
CdO/9w
CdO/CL/6w+21d
Bacillota
Bacteroidota
Pseudomonadota
Other
B
Relative abundance
100%
90%
80%
70%
60%
50%
40%
30%
20%
10%
0%
KCd/6w+1d
CdO/6w+1d
CdO/CL/6w+1d
KCd/9w
CdO/9w
CdO/CL/6w+21d
Bacillota
Bacteroidota
Other

Figure S2

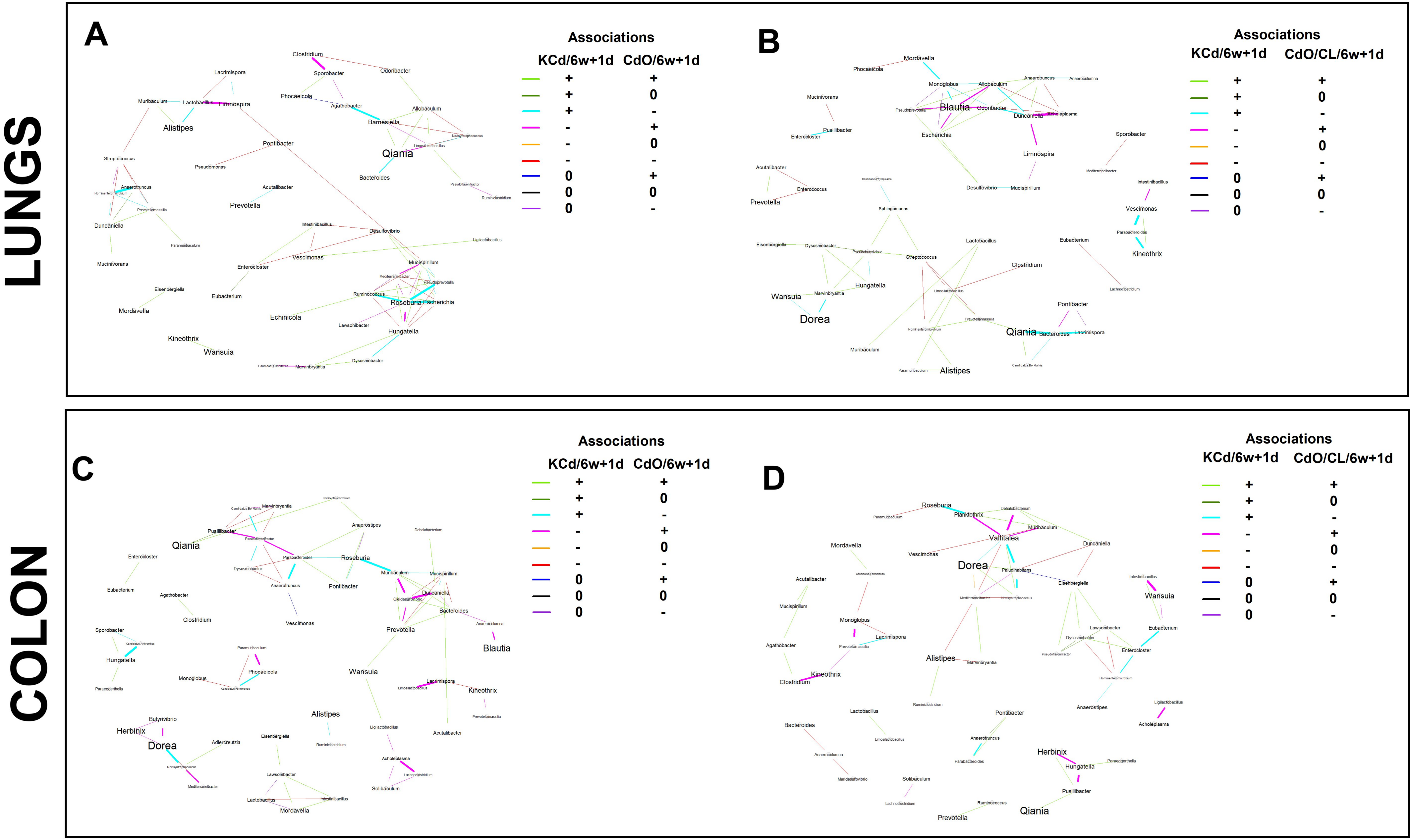
LUNGS
COLON
A
Associations
KCd/6w+1d CdO/6w+1d
+ +
+ 0
+ -
- +
- 0
- -
0 +
0 0
0 -
B
Associations
KCd/6w+1d CdO/CL/6w+1d
+ +
+ 0
+ -
- +
- 0
- -
0 +
0 0
0 -
C
Associations
KCd/6w+1d CdO/6w+1d
+ +
+ 0
+ -
- +
- 0
- -
0 +
0 0
0 -
D
Associations
KCd/6w+1d CdO/CL/6w+1d
+ +
+ 0
+ -
- +
- 0
- -
0 +
0 0
0 -

Figure S3

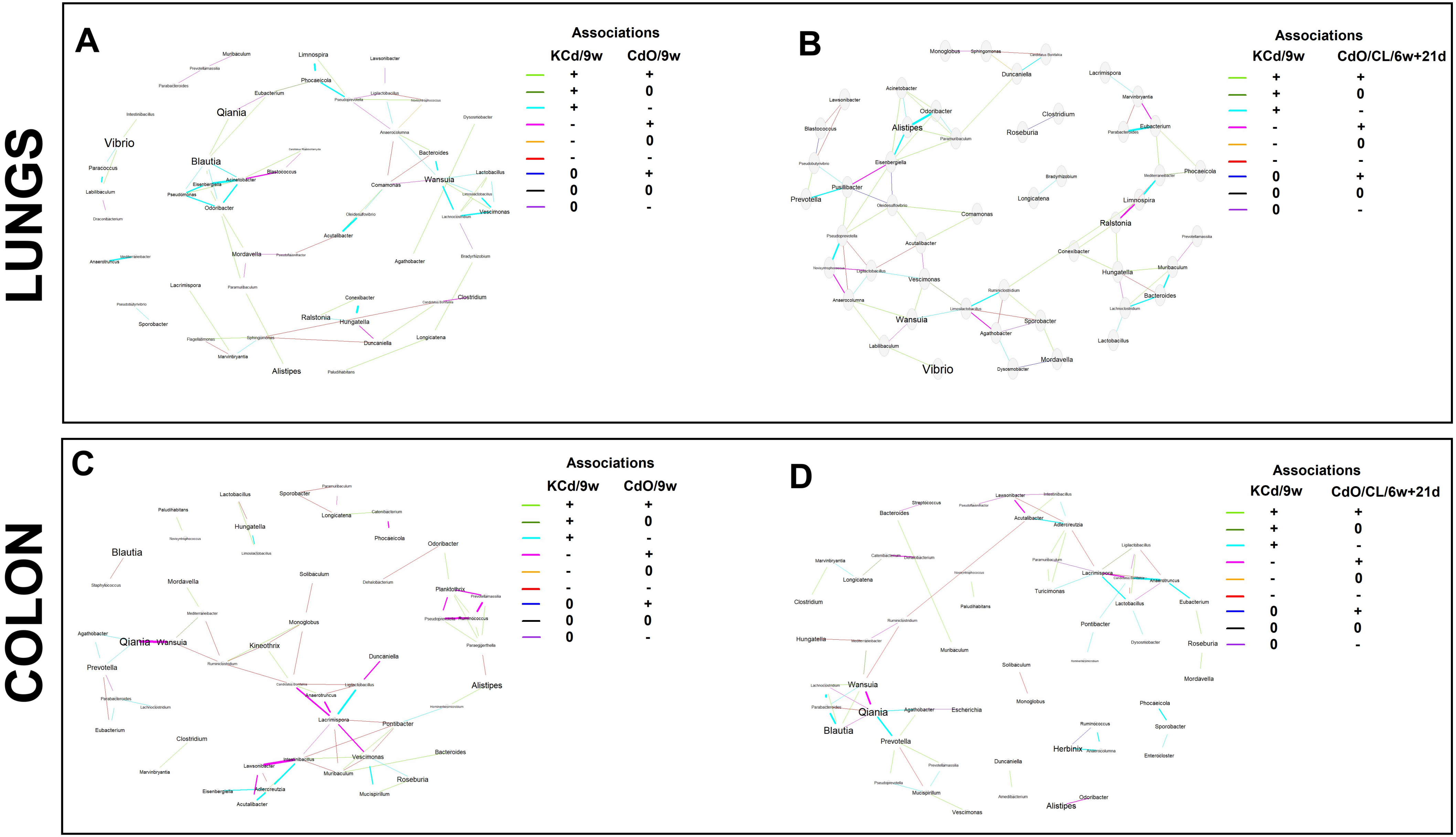
LUNGS
COLON
A
Associations
KCd/9w
CdO/9w
+ +
+ 0
+ -
- +
- 0
- -
0 +
0 0
0 -
Qiania
Vibrio
Blautia
Wansuia
Alistipes
B
Associations
KCd/9w
CdO/CL/6w+21d
+ +
+ 0
+ -
- +
- 0
- -
0 +
0 0
0 -
Alistipes
Prevotella
Wansuia
Vibrio
C
Associations
KCd/9w
CdO/9w
+ +
+ 0
+ -
- +
- 0
- -
0 +
0 0
0 -
Blautia
Qiania
Wansuia
Prevotella
Alistipes
D
Associations
KCd/9w
CdO/CL/6w+21d
+ +
+ 0
+ -
- +
- 0
- -
0 +
0 0
0 -
Wansuia
Qiania
Blautia
Prevotella
Herbinix
Alistipes

Figure S4

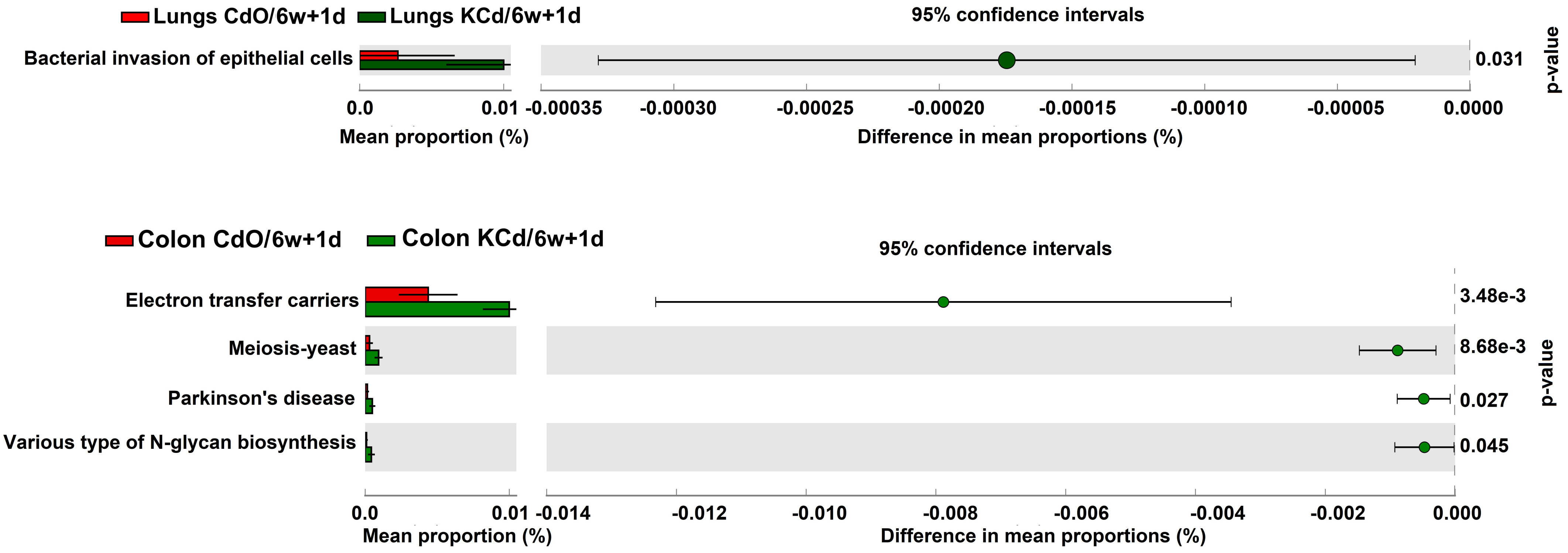

Lungs CdO/6w+1d
Lungs KCd/6w+1d
95% confidence intervals
Bacterial invasion of epithelial cells
0.031
p-value
0.0
0.01
-0.00035
-0.00030
-0.00025
-0.00020
-0.00015
-0.00010
-0.00005
0.0000
Mean proportion (%)
Difference in mean proportions (%)
Colon CdO/6w+1d
Colon KCd/6w+1d
95% confidence intervals
Electron transfer carriers
Meiosis-yeast
Parkinson's disease
Various type of N-glycan biosynthesis
3.48e-3
8.68e-3
0.027
0.045
p-value
0.0
0.01
-0.014
-0.012
-0.010
-0.008
-0.006
-0.004
-0.002
0.000
Mean proportion (%)
Difference in mean proportions (%)

Figure S5

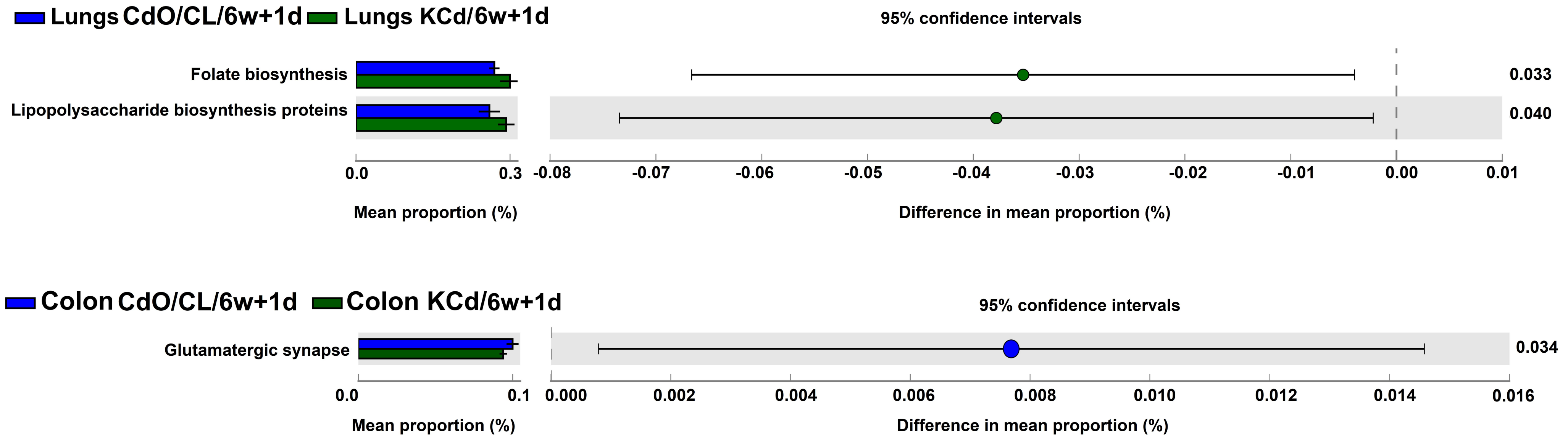

Lungs CdO/CL/6w+1d
Lungs KCd/6w+1d
95% confidence intervals
Folate biosynthesis
Lipopolysaccharide biosynthesis proteins
0.033
0.040
0.0
0.3
-0.08
-0.07
-0.06
-0.05
-0.04
-0.03
-0.02
-0.01
0.00
0.01
Mean proportion (%)
Difference in mean proportion (%)
Colon CdO/CL/6w+1d
Colon KCd/6w+1d
95% confidence intervals
Glutamatergic synapse
0.034
0.0
0.1
0.000
0.002
0.004
0.006
0.008
0.010
0.012
0.014
0.016
Mean proportion (%)
Difference in mean proportion (%)

Figure S6

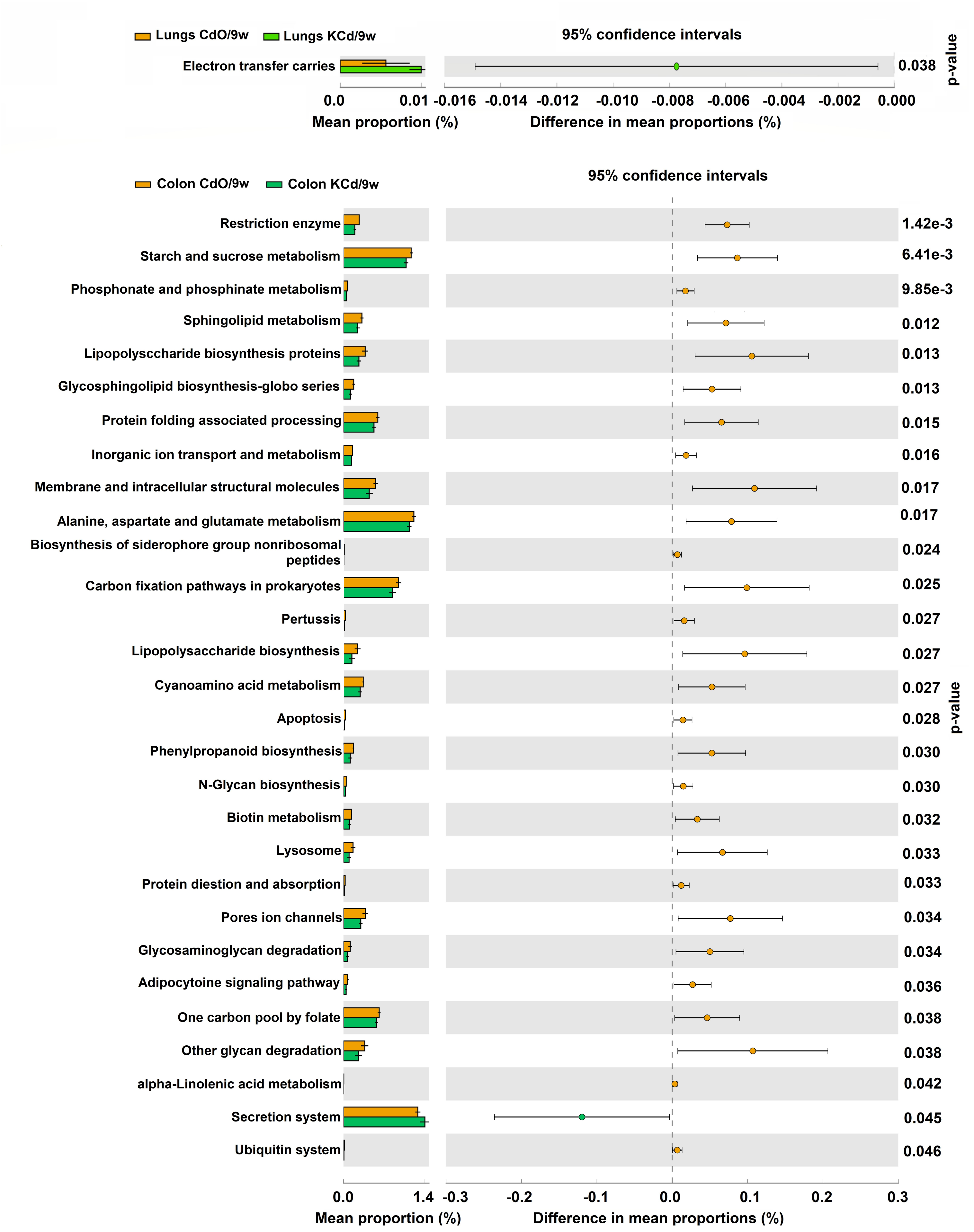

Lungs CdO/9w
Lungs KCd/9w
95% confidence intervals
Electron transfer carries
0.038
p-value
0.0
0.01
-0.016 -0.014 -0.012 -0.010 -0.008 -0.006 -0.004 -0.002 0.000
Mean proportion (%)
Difference in mean proportions (%)
Colon CdO/9w
Colon KCd/9w
95% confidence intervals
Restriction enzyme 1.42e-3
Starch and sucrose metabolism 6.41e-3
Phosphonate and phosphinate metabolism 9.85e-3
Sphingolipid metabolism 0.012
Lipopolysccharide biosynthesis proteins 0.013
Glycosphingolipid biosynthesis-globo series 0.013
Protein folding associated processing 0.015
Inorganic ion transport and metabolism 0.016
Membrane and intracellular structural molecules 0.017
Alanine, aspartate and glutamate metabolism 0.017
Biosynthesis of siderophore group nonribosomal peptides 0.024
Carbon fixation pathways in prokaryotes 0.025
Pertussis 0.027
Lipopolysaccharide biosynthesis 0.027
Cyanoamino acid metabolism 0.027
Apoptosis 0.028
Phenylpropanoid biosynthesis 0.030
N-Glycan biosynthesis 0.030
Biotin metabolism 0.032
Lysosome 0.033
Protein diestion and absorption 0.033
Pores ion channels 0.034
Glycosaminoglycan degradation 0.034
Adipocytoine signaling pathway 0.036
One carbon pool by folate 0.038
Other glycan degradation 0.038
alpha-Linolenic acid metabolism 0.042
Secretion system 0.045
Ubiquitin system 0.046
p-value
0.0
1.4
-0.3 -0.2 -0.1 0.0 0.1 0.2 0.3
Mean proportion (%)
Difference in mean proportions (%)

Figure S7

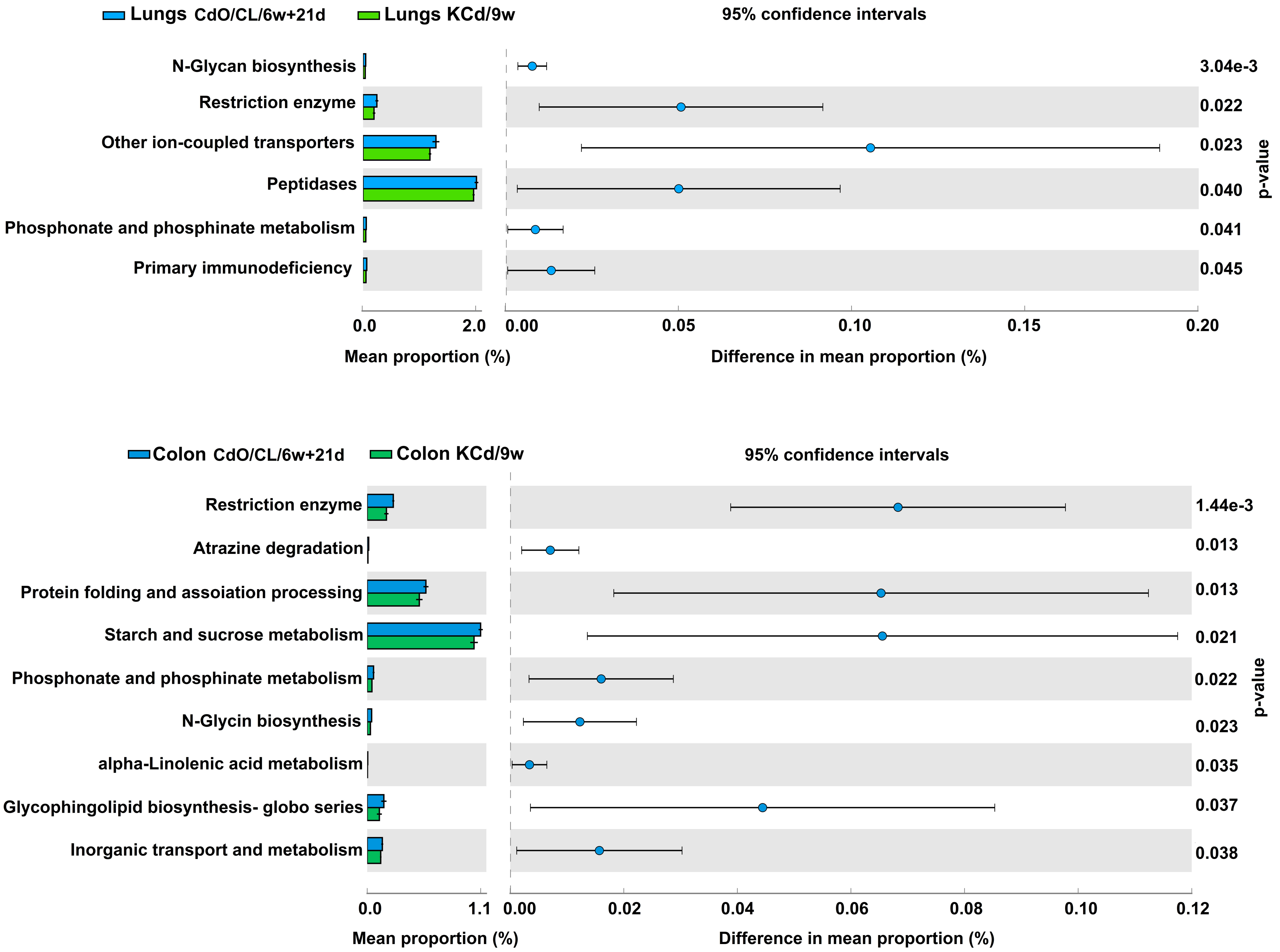

Lungs CdO/CL/6w+21d
Lungs KCd/9w
95% confidence intervals
N-Glycan biosynthesis
Restriction enzyme
Other ion-coupled transporters
Peptidases
Phosphonate and phosphinate metabolism
Primary immunodeficiency
3.04e-3
0.022
0.023
0.040
0.041
0.045
p-value
0.0
2.0
0.00
0.05
0.10
0.15
0.20
Mean proportion (%)
Difference in mean proportion (%)
Colon CdO/CL/6w+21d
Colon KCd/9w
95% confidence intervals
Restriction enzyme
Atrazine degradation
Protein folding and assoiation processing
Starch and sucrose metabolism
Phosphonate and phosphinate metabolism
N-Glycin biosynthesis
alpha-Linolenic acid metabolism
Glycophingolipid biosynthesis- globo series
Inorganic transport and metabolism
1.44e-3
0.013
0.013
0.021
0.022
0.023
0.035
0.037
0.038
p-value
0.0
1.1
0.00
0.02
0.04
0.06
0.08
0.10
0.12
Mean proportion (%)
Difference in mean proportion (%)
